# Advances in isoxazole chemistry and their role in drug discovery

**DOI:** 10.1039/d4ra08339c

**Published:** 2025-03-17

**Authors:** Glanish Jude Martis, Santosh L. Gaonkar

**Affiliations:** a Department of Chemistry, Manipal Institute of Technology, Manipal Academy of Higher Education Manipal 576104 Karnataka India sl.gaonkar@manipal.edu

## Abstract

Isoxazoles are a class of five-membered heterocyclic compounds that have gained significant attention in medicinal chemistry due to their diverse biological activities and therapeutic potential. Recent advances in isoxazole chemistry have led to the development of novel synthetic strategies, enabling the creation of a wide array of isoxazole derivatives with enhanced bioactivity and selectivity. This review explores the latest progress in isoxazole synthesis, highlighting key methodologies such as transition metal-catalyzed cycloadditions, green chemistry approaches, and regioselective functionalization techniques. These advances have not only improved the efficiency of isoxazole synthesis but have also facilitated the design of more complex and bioactive derivatives. In addition to their synthetic advances, isoxazoles have demonstrated a broad spectrum of biological activities, including antimicrobial, anticancer, anti-inflammatory, and neuroprotective effects, making them attractive candidates in drug discovery. This review discusses the structural modifications that enhance their pharmacological properties and their potential for developing therapies for diseases such as cancer, neurodegenerative disorders, and infections. Moreover, we examine the emerging trends in isoxazole-based drug discovery, such as the development of multi-targeted therapies and personalized medicine approaches. The evolving role of isoxazoles in drug discovery underscores their continued importance in modern pharmaceutical research and their potential to address unmet medical needs.

## Abstract 2

Recent advances in synthetic strategies of isoxazoles and their role in medicinal chemistry.

## Graphical abstract

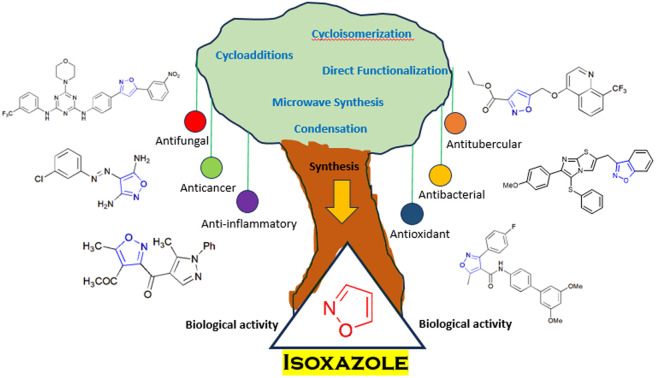

## 1 Introduction

**Fig. 1:**
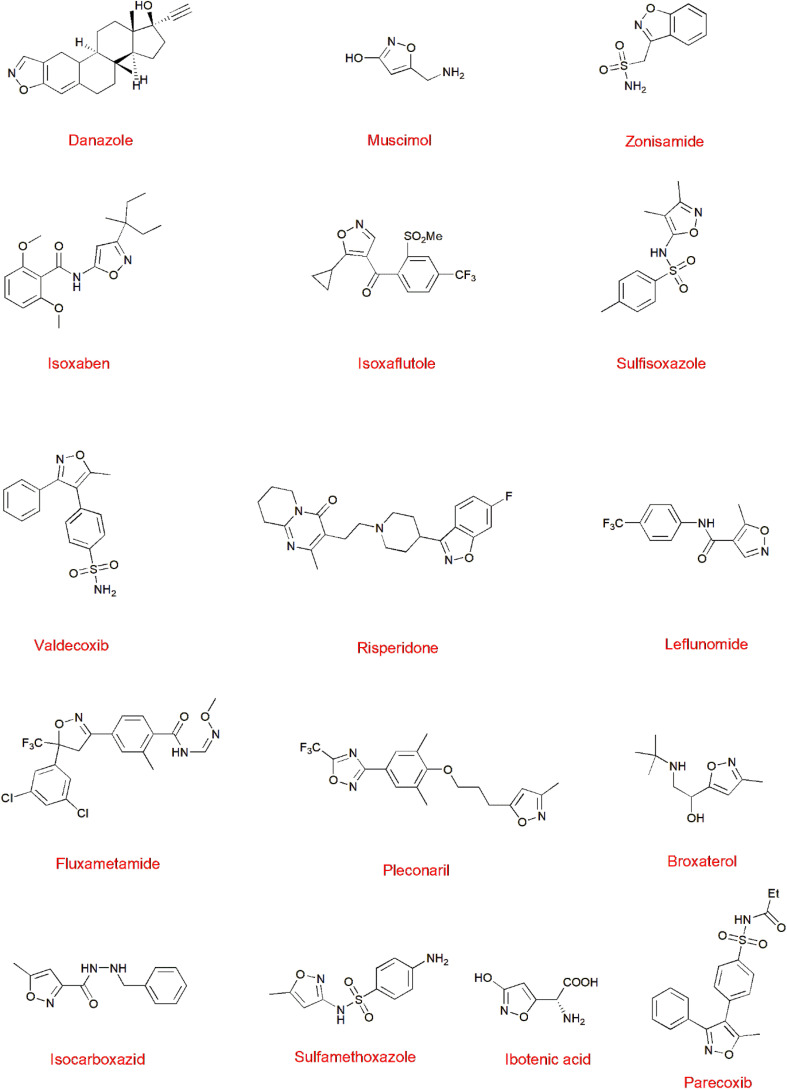
Isoxazole-containing drugs.

Isoxazole is a five-membered heterocycle with one nitrogen and oxygen connected adjacent to each other. Analogous to this, there is another structure that is partially saturated, isoxazoline. Several biologically and pharmacologically active compounds have these moieties, which make them the best candidates for medicinal diversity.^1–4^ In recent years, several strategies and methods have been developed to synthesize isoxazole/isoxazoline derivatives. The formation of various fused heterocycles from isoxazoles is noteworthy and substantial.^5^ The reason for this approach is that these derivatives possess various biological properties such as antioxidant,^6,7^ antibacterial,^8,9^ antifungal,^10,11^ anticancer,^12,13^ insecticidal,^14^ anti-inflammatory,^15,16^ antidiabetic,^17,18^ and analgesic^19^ properties. These derivatives are also effective against Alzheimer's disease.^20,21^ In particular, isoxazole has gained much attention and importance because of its electron-rich aromatic structure. Moreover, the weak nitrogen and oxygen bond is attributed to ring cleavage reactions.^22,23^ Currently, isoxazole is present not only in pharmaceuticals, but also in natural products and agrochemicals.^24^ Direct extraction of chemical constituents from plant sources results in low pharmacological effects. Thus, it is necessary for inducing structural modifications to improve their biological activity, pharmacological efficiency and drug selectivity. By doing so, it is also possible to have different activities from the parent source.^25^ Isoxazoles are found in various pharmaceutical drugs such as danazol,^26^ muscimol,^27,28^ zonisamide,^29^ isoxaben,^30^ isoxaflutole,^31^ sulfisoxazole,^32,33^ valdecoxib,^34,35^ fluxametamide,^36,37^ risperidone,^38,39^ leflunomide,^40^ pleconaril,^41,42^ broxaterol,^43^ isocarboxazid,^44^ sulfamethoxazole,^45^ ibotenic acid,^46^ and parecoxib.^47^ The substituents present on the isoxazole moiety play a vital role in complex formation, especially, when functional groups are present.^48,49^ Thus, the structure of isoxazoles has undergone several modifications and alterations to increase their biological activity.^50^ However, by including isoxazole in medicinal targets, there can be improvement in pharmacokinetic profiles, increased efficacy and decrease in toxicity.^51,52^ However, the scope of functionalized isoxazoles is wide and has delivered promising results.^53^ (Fig. 1).

## 2 Synthetic strategies for isoxazoles and their derivatives

### 2.1 Cycloaddition reactions

#### 2.1.1 1,3 Dipolar cycloaddition

**Scheme 1:**
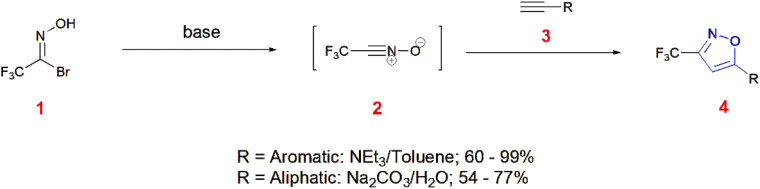
Synthesis of 3-trifluoromethyl-5-substituted isoxazole derivatives 4.

Many heterocycles today are the result of cycloaddition reactions. Among such molecules, isoxazoles result from 1,3 dipolar cycloaddition reactions. Similarly, Ley and co-workers synthesized 3-trifluoromethylisoxazoles 4 from hydroximoyl bromide 1. The dipole nitrile oxide 2 is formed from the precursor hydroxymoyl bromide 1 and this dipole undergoes cycloaddition with the substituted terminal alkynes 3 to give 3-trifluoromethyl-5-substituted isoxazole derivatives 4. The combination of the solvent systems in association with suitable bases significantly increased the yield and accelerated the cycloadditions. Triethylamine/toluene acts as a good system when phenylacetylene, 4-bromophenyl acetylene, 4-methoxy acetylene are used and sodium carbonate/water is used for cyclopropyl acetylene and cyclopentylacetylene.^54^ (Scheme 1).

**Scheme 2:**
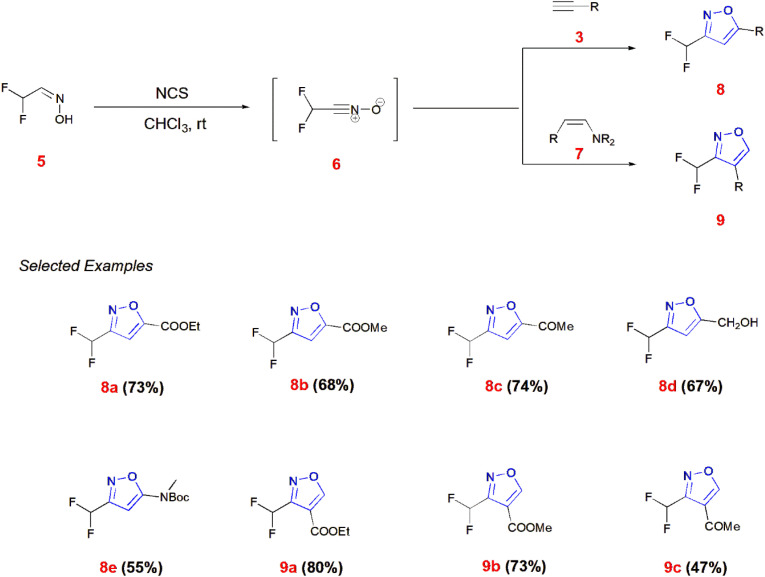
Synthesis of 3-difluoromethyl isoxazole derivatives 8 and 9.

Mykhailiuk and co-workers generated difluoromethyl isoxazoles 8 and 9 from difluoro oxime 5 reacting with *N*-chloro-succinimide (NCS) in the presence of chloroform as solvent to give the dipole difluoro nitrile oxide 6. This dipole 6 reacts with terminal alkynes 3 and enamines 7 to give 3,5-disubstituted 8 and 3,4-disubstituted isoxazoles 9, respectively.^55^ (Scheme 2).

**Scheme 3:**
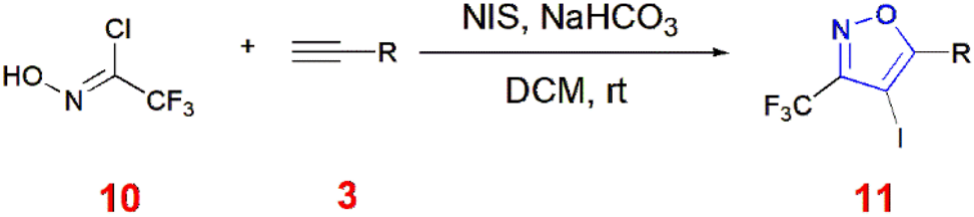
Synthesis of 3-trifluoromethyl-4-iodoisoxazole 11.

Wu *et al.*, synthesized 3-trifluoromethyl-4-iodoisoxazoles 11 from trifluoroacetohydroximoyl chloride 10, terminal alkynes 3 and *N*-iodosuccinimide in a one-pot reaction containing sodium bicarbonate as the base and dichloromethane as the solvent at room temperature. This reaction afforded up to 81% yield.^56^ (Scheme 3).

**Scheme 4:**
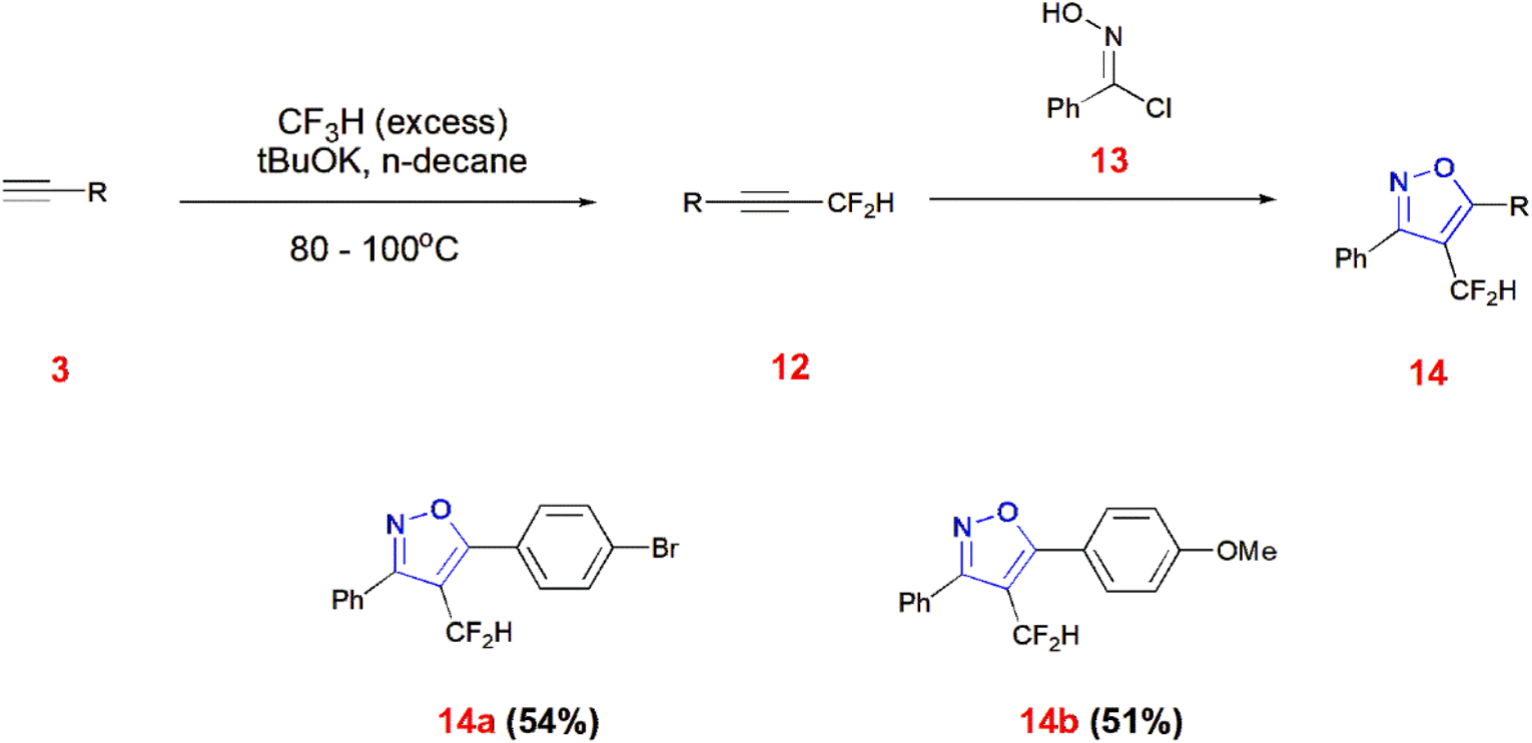
Synthesis of 4-difluoromethyl isoxazoles 14.

Shibata *et al.*, reported the synthesis of 4-difluoromethyl isoxazoles 14 from difluoromethyl alkynes 12 and imidoyl chloride 13 using triethylamine as the base with dichloromethane. Difluoromethyl alkynes 12 were obtained from terminal alkynes 3*via* reaction with fluoroform (source of difluorocarbene) in the presence of potassium *tert*-butoxide and *n*-decane heated at 80–100 °C for 3 h.^57^ (Scheme 4).

**Scheme 5:**
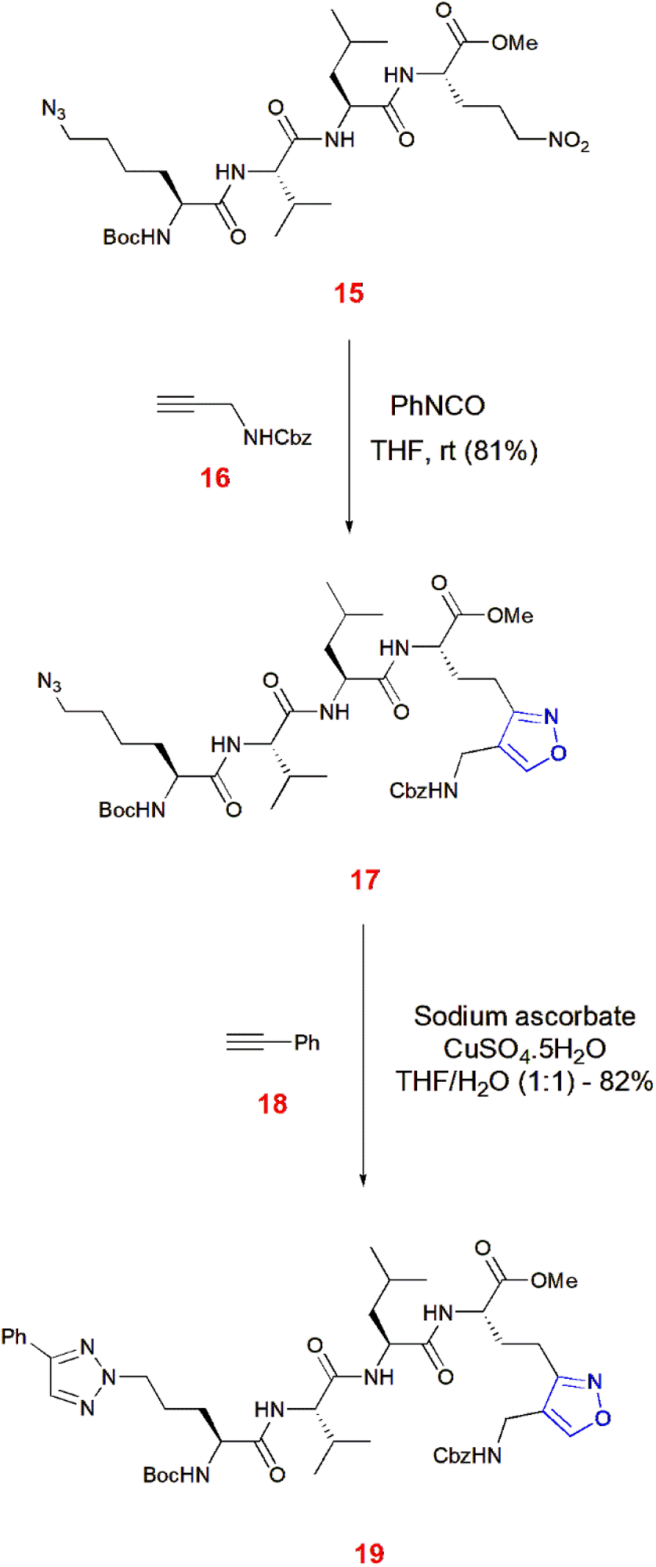
Orthogonal cycloaddition giving isoxazole–triazole peptide 19.

Gopi and co-workers illustrated an orthogonal cycloaddition reaction leading to the formation of doubly conjugated peptide 17 bearing isoxazole and triazole moieties. First, nitro-alkane tethered peptide 15 was treated with phenyl isocyanate and *N*-Cbz-propargylamine 16 in THF at room temperature to yield 81% of the peptide containing isoxazole 17. This isoxazole scaffold 17 was made to undergo alkyne–azide cycloaddition with phenylacetylene 18 in the presence of sodium ascorbate, CuSO_4_·5H_2_O in equimolar THF/water to yield 82% of triazole–isoxazole peptide 19.^58^ (Scheme 5).

**Scheme 6:**
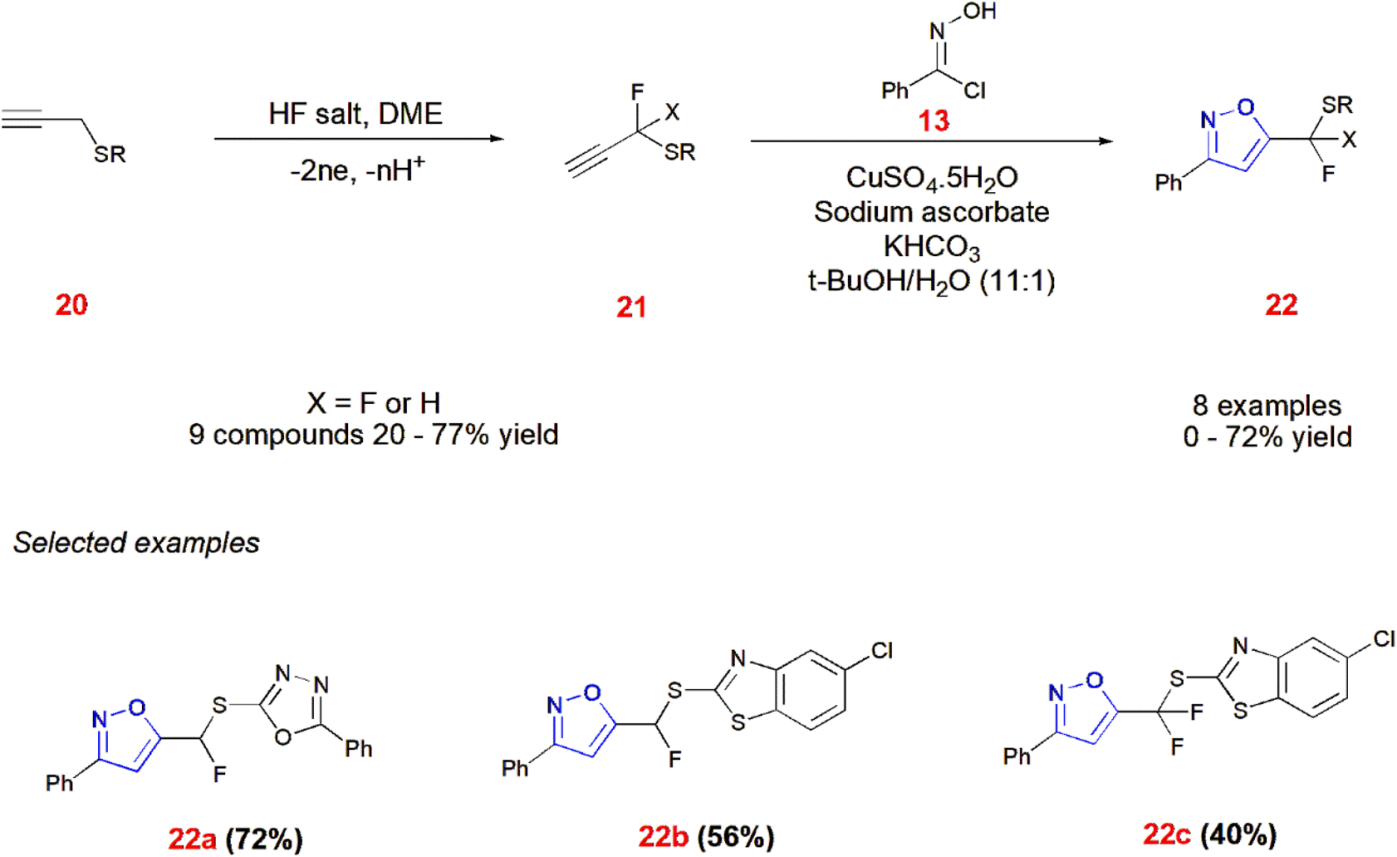
Electrochemical fluorination and Cu catalyzed cycloaddition giving isoxazoles 22.

Nitrile oxides obtained from imidoyl chloride 13 undergo 1,3-dipolar cycloaddition of mono- or di-fluorinated propargyl thioethers 21 catalyzed by a copper sulfate/sodium ascorbate line to give isoxazoles 22. First, propargyl thioethers 20 are electrochemically reacted with HF salt to give partially fluorinated terminal alkynes 21.^59^ (Scheme 6).

**Scheme 7:**
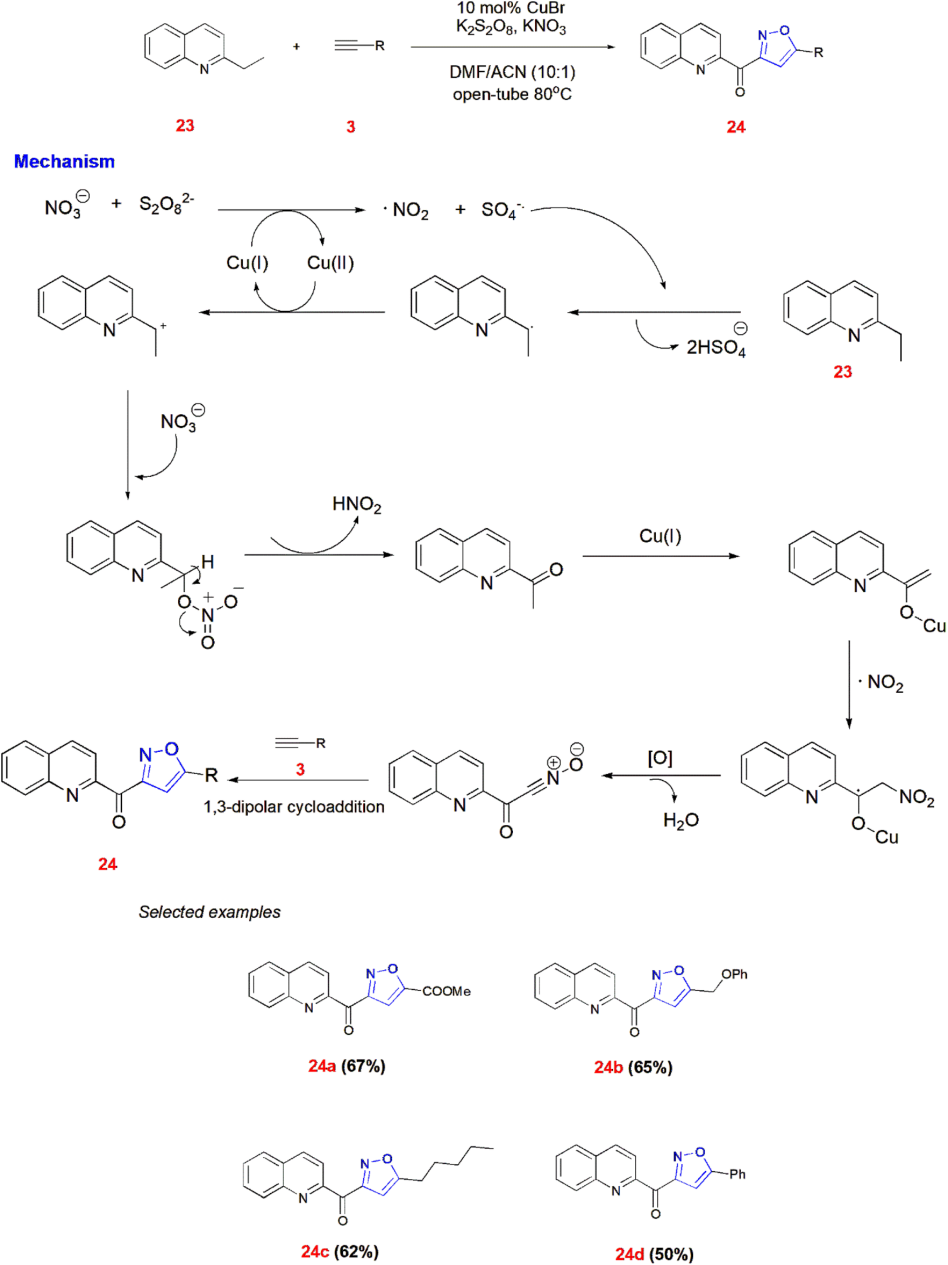
Cu-catalyzed synthesis of quinoline–isoxazoles 24.

Yang *et al.*, demonstrated the generation of nitrile oxide from 2-ethylazaarene 23, KNO_3_ and K_2_SO_8_ by selective oxidation. Then it underwent copper-catalyzed 1,3-dipolar cycloaddition reaction with terminal alkynes 3 to give quinoline–isoxazole derivatives 24. The plausible mechanism shows the role of KNO_3_ and K_2_SO_8_ in giving products along with Cu catalyst.^60^ (Scheme 7).

**Scheme 8:**
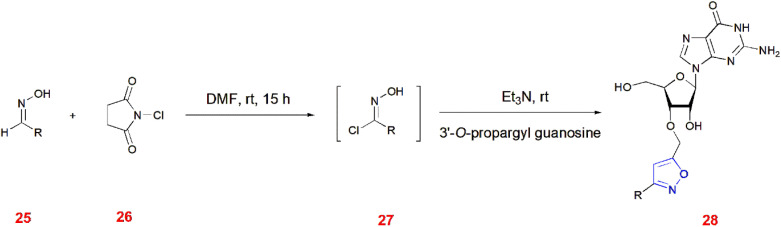
Synthesis of 3-*O*-propargyl guanosyl derived isoxazoles 28.

Kore and co-workers illustrated the 1,3-dipolar cycloaddition of 3′-*O*-propargyl guanosine with chloroximes 27 to give isoxazoles 28 in the presence of triethylamine at room temperature. Prior to this, different aldoximes 25 were chlorinated with *N*-chlorosuccinimide 26 in DMF at room temperature to generate chloroximes 27. The different derivatives and their yields are given in Table 1.^61^ (Scheme 8).

**Table 1:** Different derivatives of isoxazoles 28

R	Yield (%)
Phenyl	84
4-Methoxy-phenyl	74
9-Anthranyl	72
3-Pyridyl	77
3-Indolyl	79
2-Chlorophenyl	70
4-Nitrophenyl	77
4-Bromo-2-thiophenyl	70

#### 2.1.2 [3 + 2] cycloaddition

**Scheme 9:**
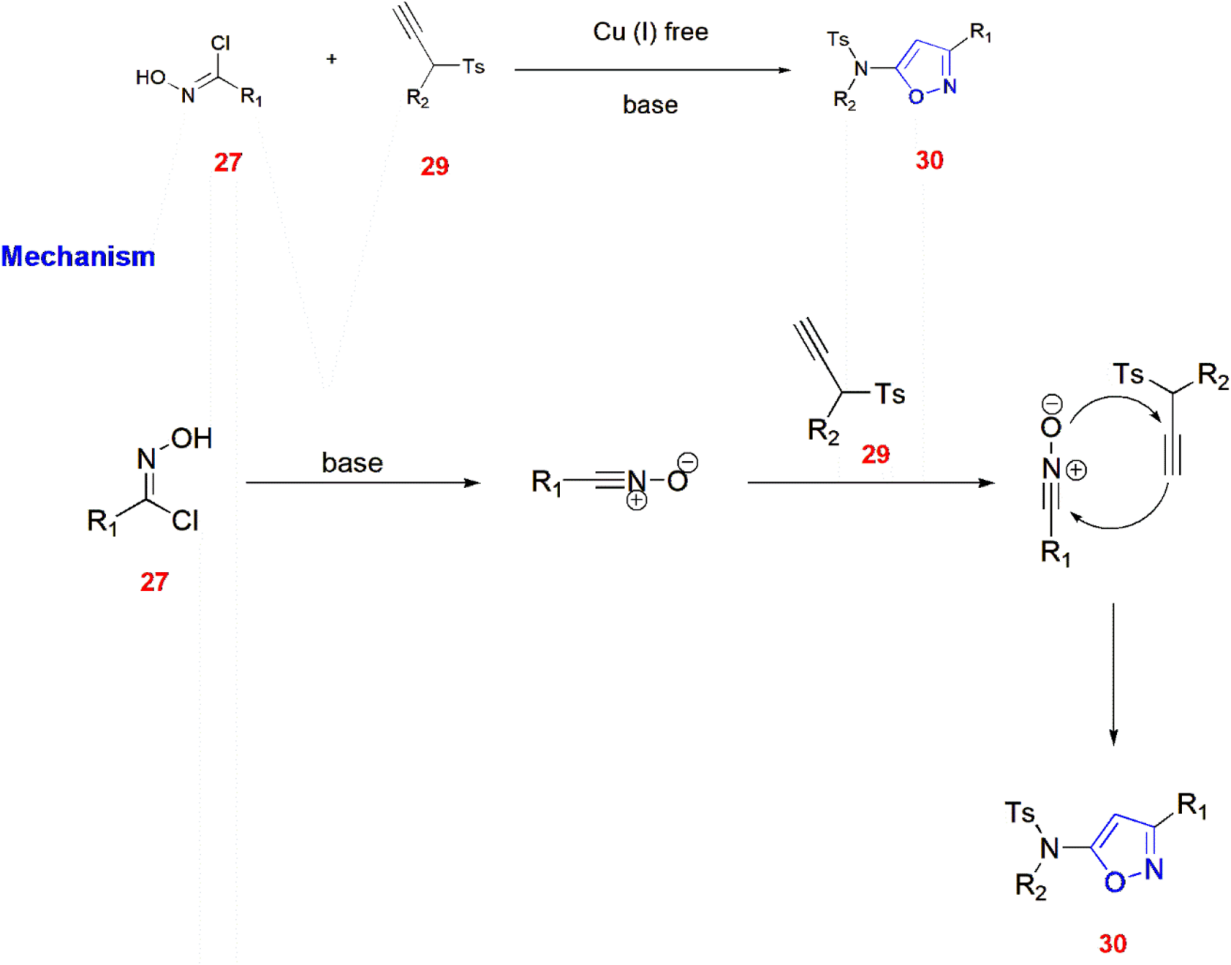
Synthesis of isoxazoles 30 from nitrile oxides and ynamides 29.

Chen and co-workers reported a method of Cu(i)-free [3 + 2] cycloaddition between nitrile oxide and electron-rich terminal ynamides 29 to give 3,5-disubstituted isoxazoles 30 with proposed mechanism. Nitrile oxides are generated from chloroximes 27 upon treatment with sodium carbonate.^62^ (Scheme 9).

**Scheme 10:**
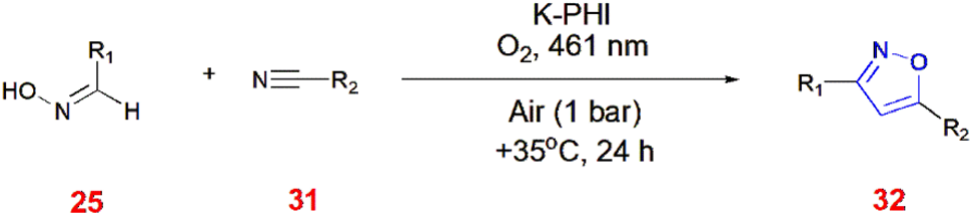
Synthesis of isoxazoles 32 by artificial photosynthesis.

Isoxazoles were generated from potassium poly(heptazine imide) (K-PHI) after artificial photosynthesis to O_2_ by maintaining a pressure of 1 bar at 461 nm. Next, the aldoximes 25 were quenched with ^1^O_2_ to form nitrile oxides, which react with alkyl nitriles 31 in a [3 + 2] cycloaddition fashion to give isoxazoles 32.^63^ (Scheme 10).

**Scheme 11:**
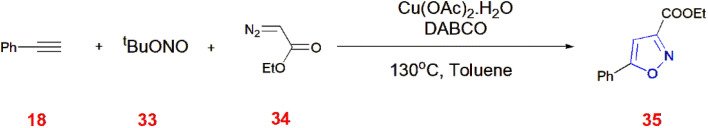
Synthesis of 3,5-disubstituted isoxazoles 35*via* [3 + 2] cycloaddition using DABCO.

Zhao *et al.*, proposed another method involving copper catalyzed [3 + 2] cycloaddition of phenylacetylene 18 with nitrile oxides derived from nitroso radical and copper carbene. A three component reaction of 18 with *tert*-butyl nitrite 33 and ethyl diazoacetate 34 in the presence of Cu(OAc)_2_·H_2_O as catalyst and 1,4-diazabicyclo[2.2.2]octane (DABCO) as base in toluene at 130 °C gave 3,5-disubstituted isoxazole 35.^64^ (Scheme 11).

#### 2.1.3 [2 + 1 + 1 + 1] cycloaddition

**Scheme 12:**
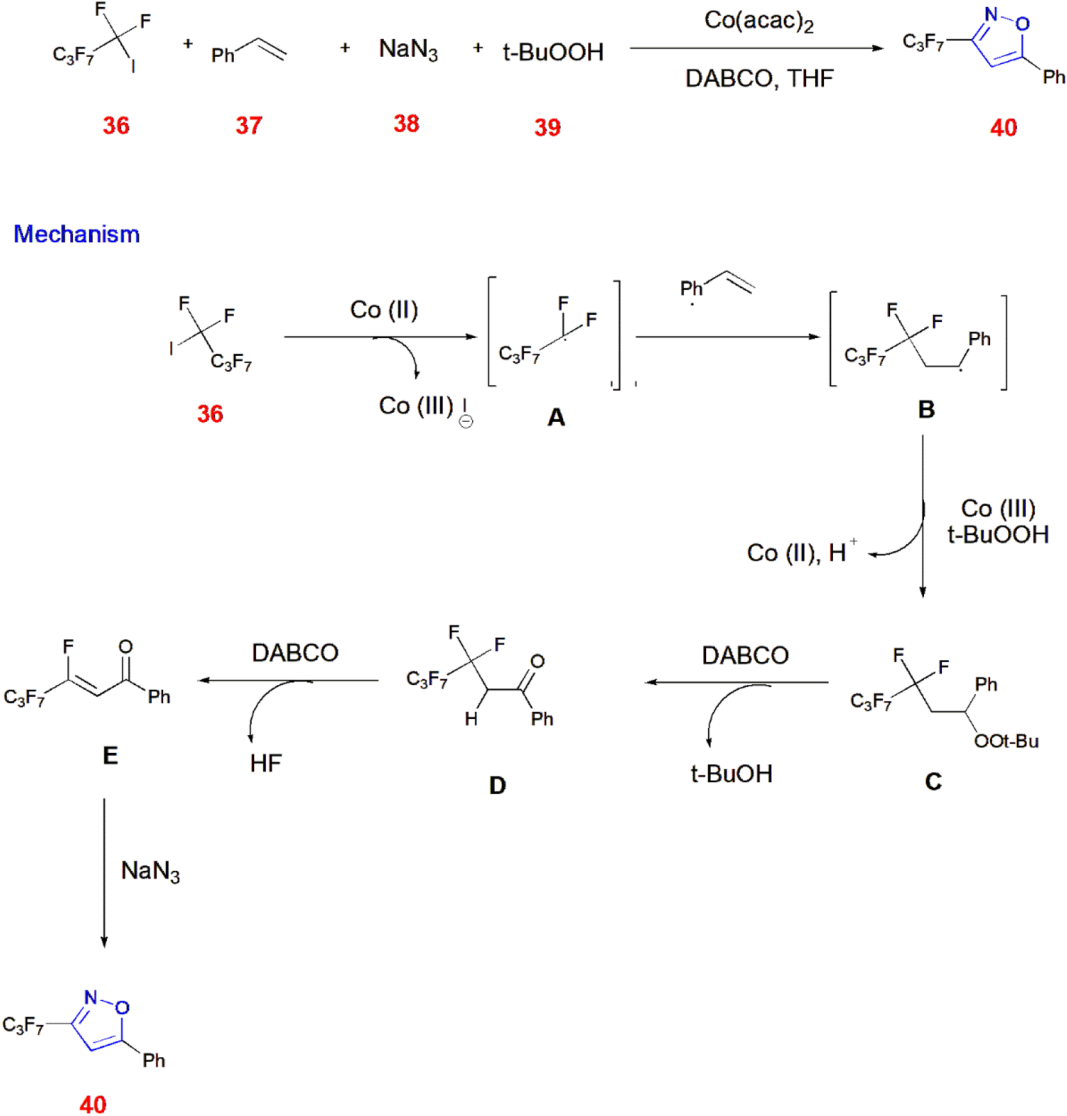
Synthesis of perfluoroalkyl isoxazole 40*via* [2 + 1 + 1 + 1] annulation.

Chen *et al.*, reported a multicomponent reaction involving [2 + 1 + 1 + 1] cycloaddition where fluorobutyl iodide 36 reacts with the catalyst Co(ii) to generate corresponding radical A, which acts upon styrene 37 to give another radical B. Coupling this radical B with *tert*-butyl peroxy radical 39 gives *β*-difluoro peroxide C. The Kornblum–DeLaMare rearrangement of this peroxide C catalyzed by DABCO led to the formation of an intermediate D having a carbonyl group that undergoes DABCO-promoted HF elimination to give unsaturated compound E. This unsaturated compound when treated with sodium azide 38, gives perfluoroalkyl isoxazole ring 40.^65^ (Scheme 12).

**Scheme 13:**
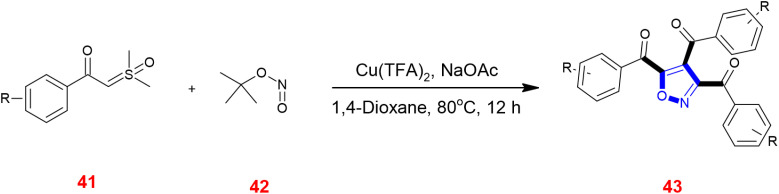
Synthesis of isoxazoles 43 from sulfoxonium ylides 41.

Tang *et al.*, annulated sulfoxonium ylides 41 with *tert*-butyl nitrite 42 catalyzed by Cu(TFA)_2_ with sodium acetate as the base and dioxane as the solvent heated at 80 °C for 12 h to give isoxazoles 43. This was one of the novel preparations of isoxazole cores involving [2 + 1 + 1 + 1] cycloaddition.^66^ (Scheme 13).

### 2.2 Condensation reactions

**Scheme 14:**
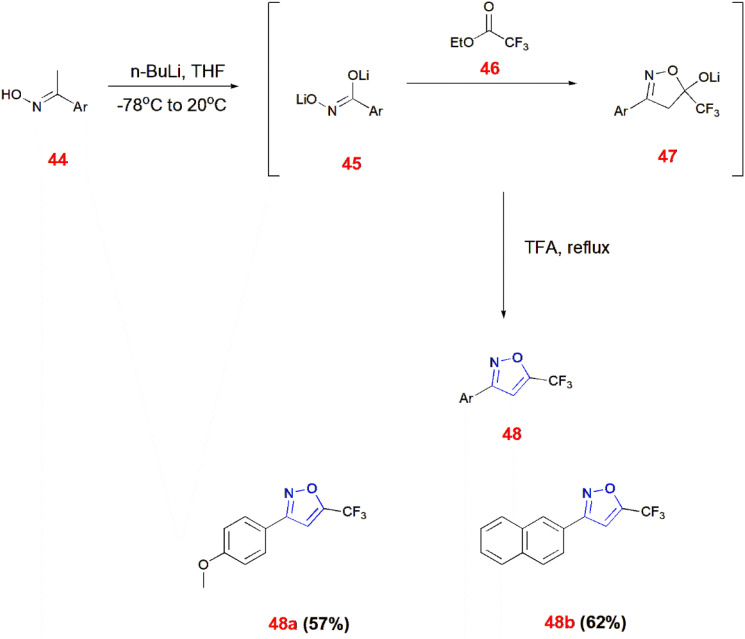
Synthesis of 5-trifluoromethyl isoxazoles 48.

Langer and co-workers synthesized 5-trifluoromethyl isoxazoles 48 from hydrazone dianions **45**, which were prepared from the reaction of *n*-BuLi with oximes 44. These dianions were then treated with trifluoroacetate 46 to give 5-trifluoromethyl isoxazoles 48*via*47 through reflux.^67^ (Scheme 14).

**Scheme 15:**
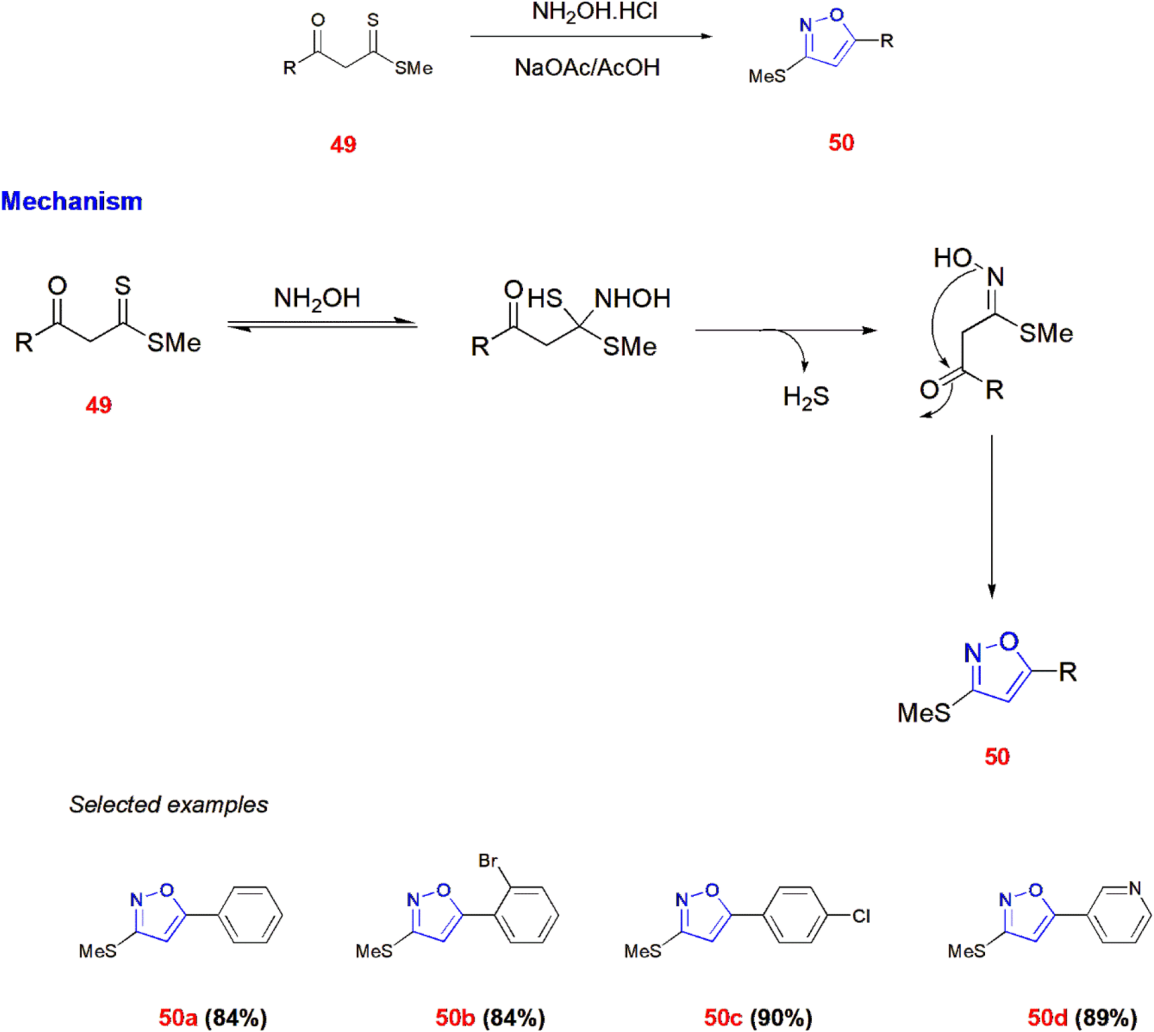
Synthesis of 3-methylthio-5aryl-isoxazoles 50.

Several 3-methylthio-5aryl-isoxazoles 50 were synthesized from *β*-oxodithioesters 49 reacting with hydroxylamine hydrochloride *via* sodium acetate and acetic acid under acidic conditions at 90 °C for 2–10 h. One key point here is that the use of acetic acid is necessary for the formation of the isoxazole ring with possible mechanistic approach.^68^ (Scheme 15).

**Scheme 16:**
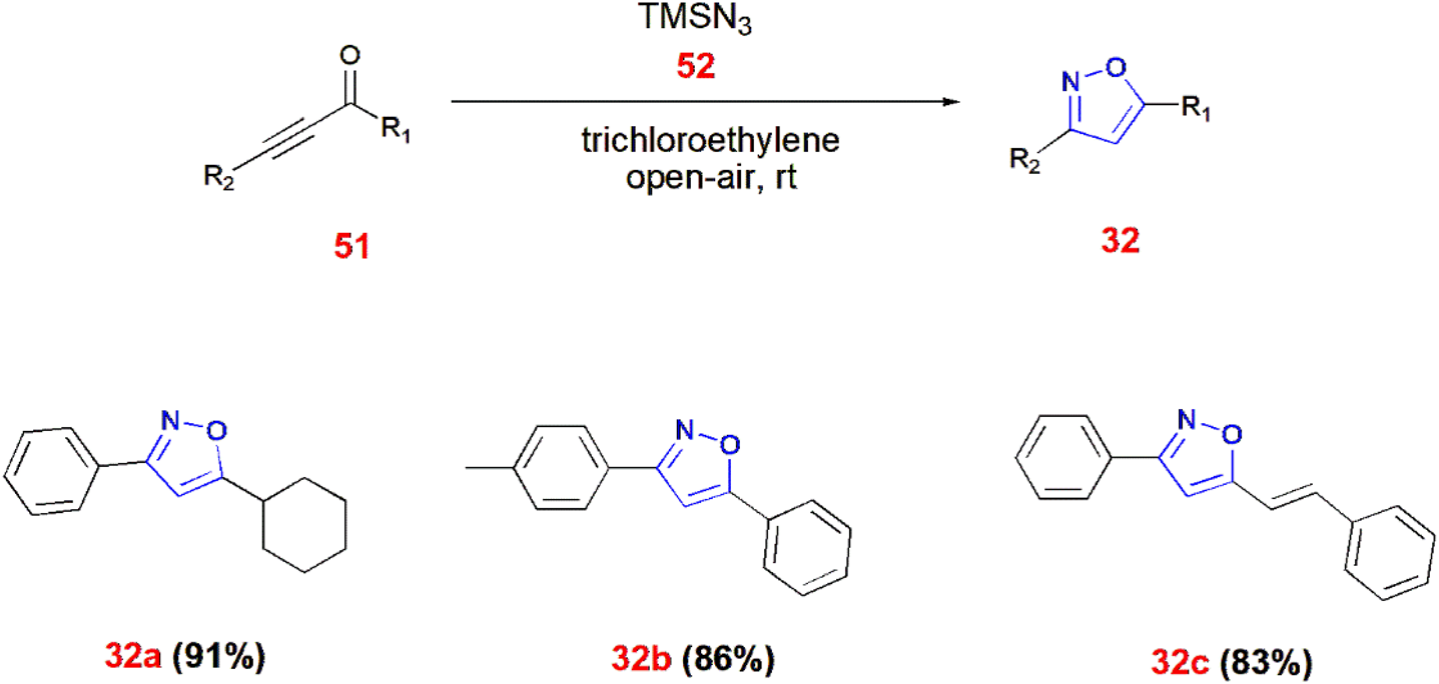
Synthesis of 3,5-disubstituted isoxazoles 32 from ynones 51.

Reddy *et al.*, proposed a synthetic strategy for 3,5-disubstituted isoxazoles 32. In this method, ynones 51 react with trimethylsilylazide 52 through *syn*-Michael addition *via* trichloroethylene in open air at room temperature to yield 3,5-disubstituted isoxazoles 32.^69^ (Scheme 16).

**Scheme 17:**
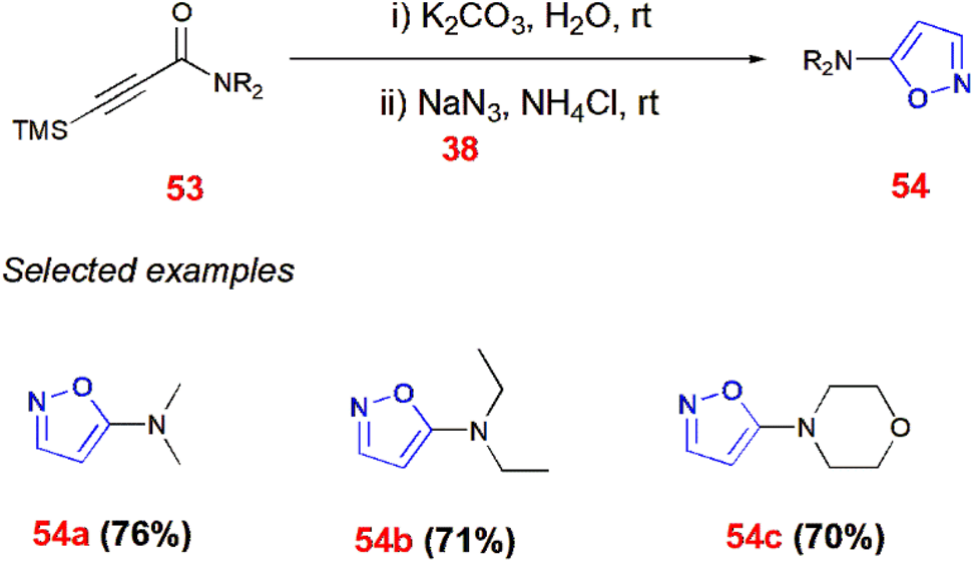
Synthesis of 5-aminoisoxazoles 54 from ynamides 53.

Similarly, ynones react with azide ion to form TMS-ynamides 53 and then react to give 5-aminoisoxazoles 54 using potassium carbonate in aqueous media at room temperature followed by the addition of sodium azide 38 in the presence of ammonium chloride. In this method, the TMS group eliminated first under basic conditions, followed by the formation of corresponding 5-aminoisoxazoles 54 from *syn*-Michael adducts.^70^ (Scheme 17).

**Scheme 18:**
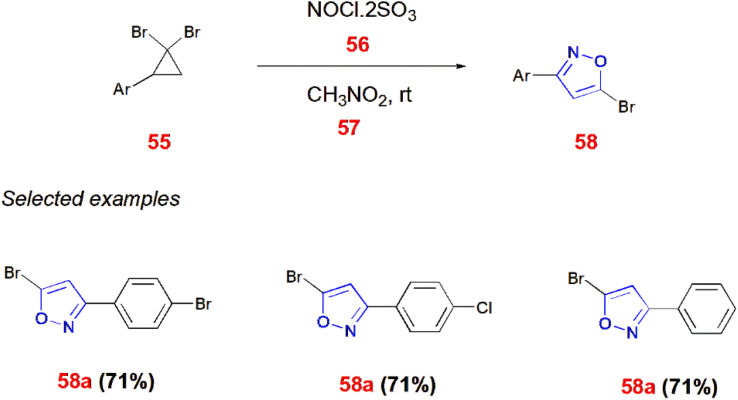
Synthesis of 5-bromoisoxazoles 58.

**Scheme 19:**
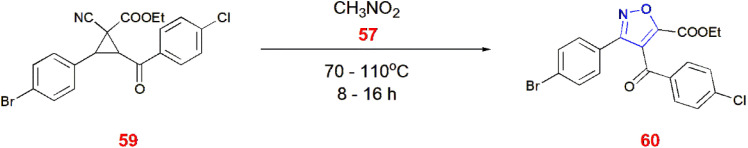
Synthesis of isoxazole-5-carboxylate 60.

Bondarenko and co-workers reported a method of producing 5-bromoisoxazoles 58. Here, 2 aryl-1,1-dibromocyclopropanes 55 undergo nitrosation with nitrosyl chloride 56 in the presence of nitromethane 57 to give 5-bromoisoxazoles 58 at room temperature.^71^ (Scheme 18) Similarly, 1,8-diazabicyclo[5.4.0]undec-7-ene (DBU)-facilitated ring opening of the aryl-cyclopropane 59 generated isoxazole-5-carboxylate 60 in the presence of nitromethane 57 as a driving force for ring cleavage maintained at 70–110 °C for 8–16 h. DBU resulted 90% of the yield with DMF as the solvent whereas acetonitrile and THF produced no yield.^72^ (Scheme 19).

### 2.3 Microwave-induced synthesis

**Scheme 20:**
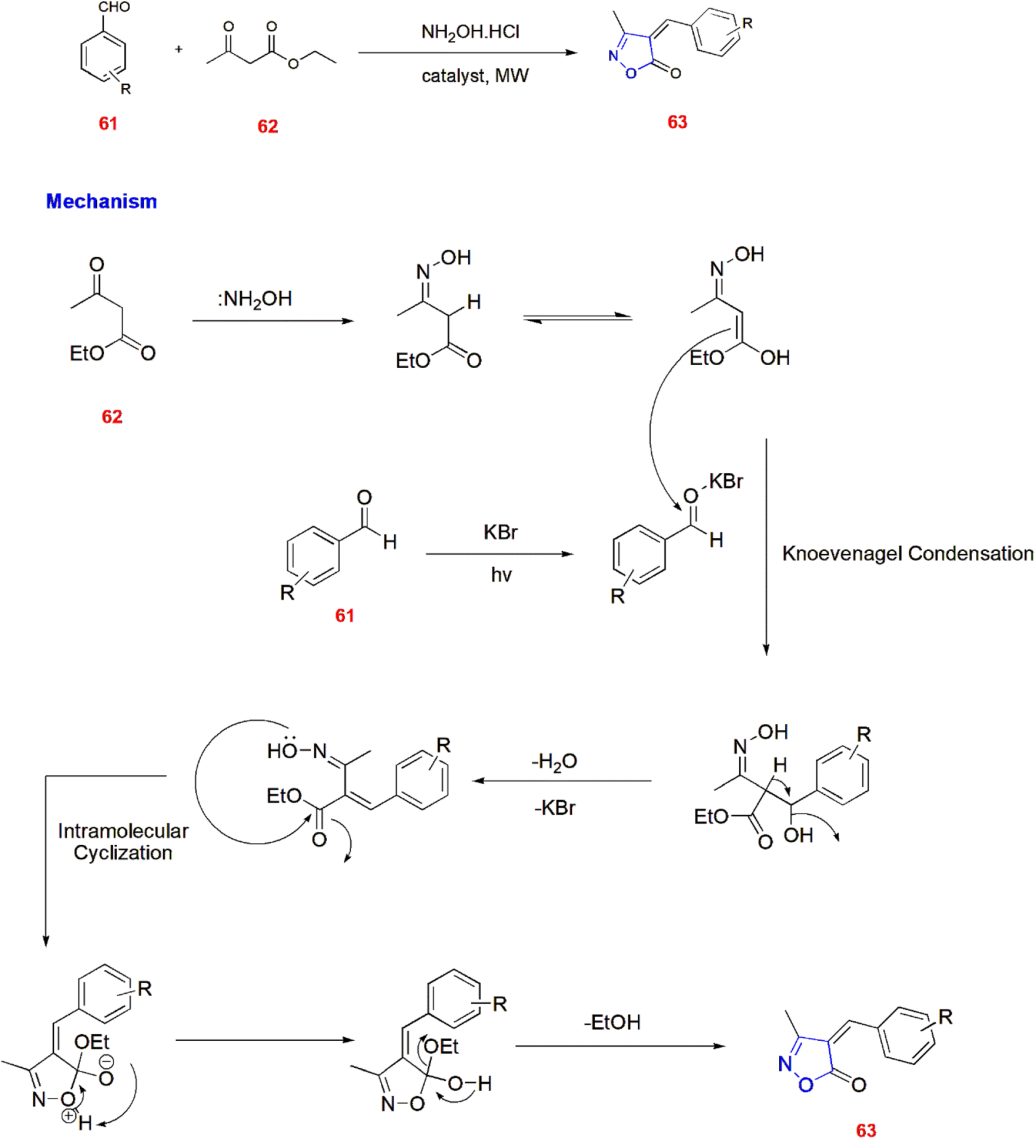
Microwave synthesis of 3,4-disubstituted isoxazole-*5*(4*H*)-ones 63.

Kulkarni developed a solvent free method for synthesizing 3,4-disubstituted isoxazole-5(4*H*)-ones 63*via* microwave-induced organic synthesis. In this method, substituted aldehydes 61 were treated with hydroxylamine hydrochloride and ethyl acetoacetate 62 in the presence of catalysts such as KBr, KCl, NaOAc, MgCl_2_*etc* and irradiated at 200 W to obtain 3,4-disubstituted isoxazole-5(4*H*)-ones 63.^73^ (Scheme 20).

**Scheme 21:**
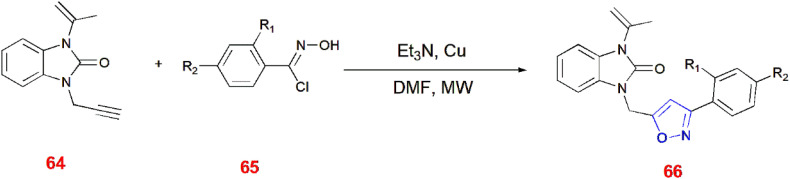
Microwave assisted synthesis of 3,5-disubstituted isoxazole 66.

Sebbar *et al.*, illustrated microwave synthesis for 3,5-disubstituted isoxazole 66 from alkyne of benzimidazole-2-one 64 and hydroxy-4-methoxy-benzene-carboximidoyl chloride 65 in the presence of triethylamine, DMF and catalyst Cu.^74^ (Scheme 21).

**Scheme 22:**
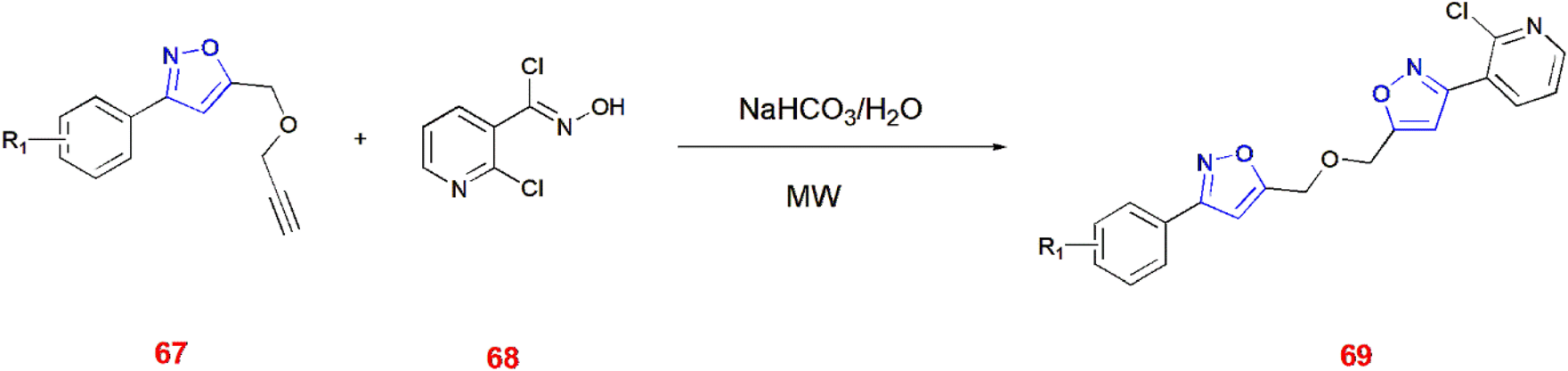
Microwave induced synthesis of 3-substituted bis isoxazole ether 69.

Qiang Gu and others gave an efficient method for synthesizing 3-substituted bis isoxazole ether 69 from chloro derived pyridyl oxime 68 and 3-substituted phenyl-5-((prop-2-yn-1-yloxy)methyl) isoxazoles 67 in the presence of sodium bicarbonate as a base and acid-binding agent in THF and aqueous media followed by microwave irradiation of the reaction mixture.^75^ (Scheme 22).

**Scheme 23:**
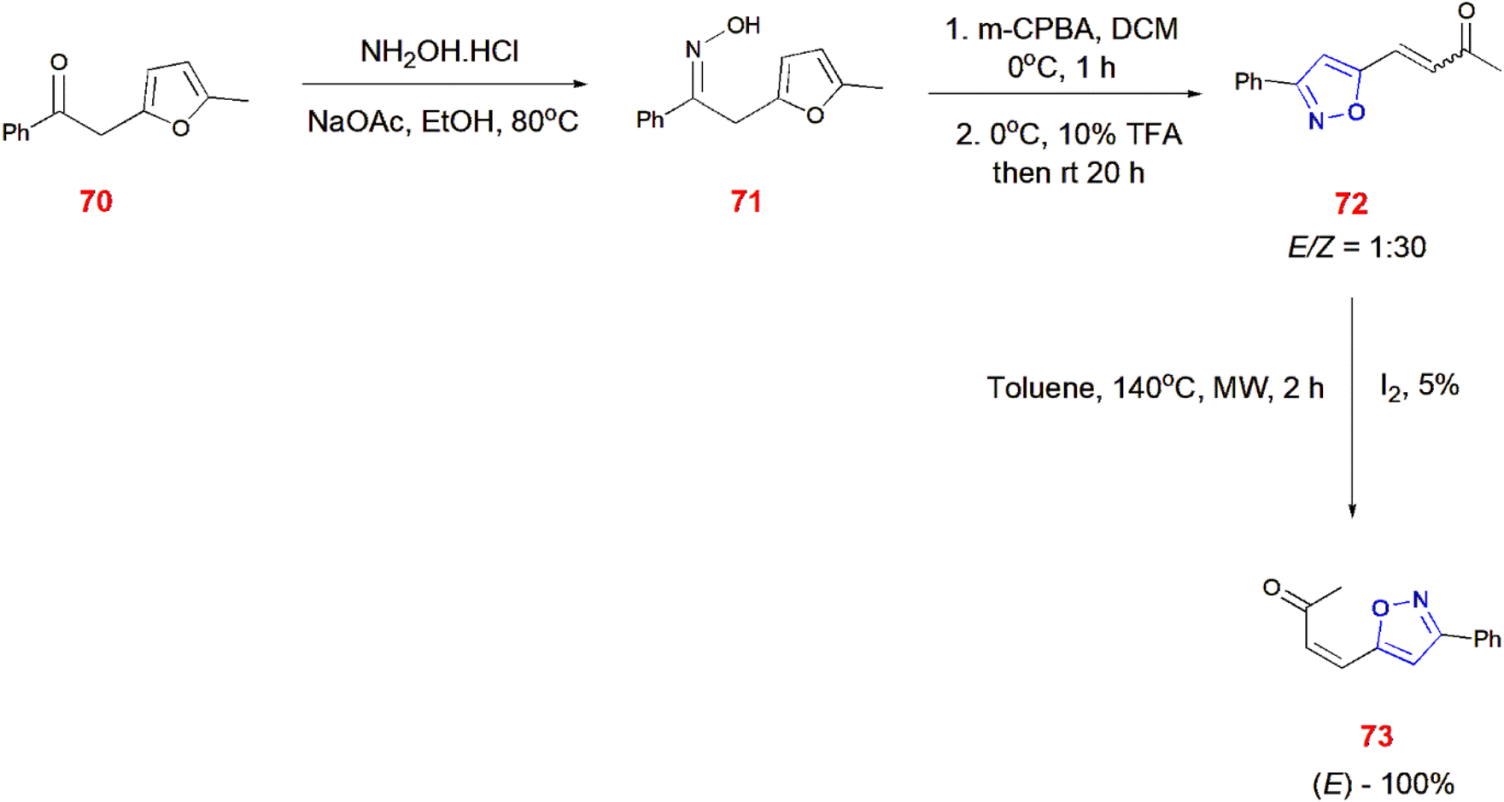
Microwave synthesis of (*E*)-4-(3-phenylisoxazol-5-yl)but-3-en-2-one 73.

Microwave synthesis was also found to be helpful in isomerization reactions. Furfuryl ketone 70 was reacted with hydroxylamine hydrochloride to form furfuryl oxime 71 which was then treated with *m*-chloroperbenzoic acid (*m*-CPBA) at 0 °C for 1 h. The addition of trifluoroacetic acid at 0 °C and then bringing to room temperature gave 4-(3-phenylisoxazol-5-yl)but-3-en-2-one 72 as a mixture of *E* and *Z* isomers at a ratio of 1 : 30. The mixture 72 was made to undergo iodine-mediated isomerization at 140 °C in the presence of toluene in microwave synthesizer to obtain the *E* form of isomer 73.^76^ (Scheme 23).

**Scheme 24:**
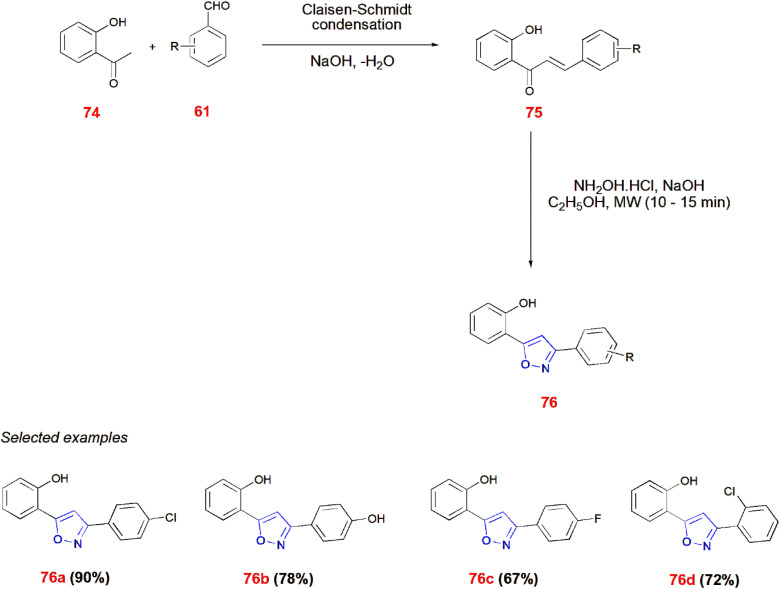
Microwave induced synthesis of isoxazole derivatives 76.

*O*-Hydroxyacetophenone 74 undergoes Claisen–Schmidt condensation with substituted aldehydes 61 in the presence of NaOH to give chalcones 75. When irradiated in Microwave with hydroxylamine hydrochloride in ethanol for 10–15 min, these chalcones 75 afforded isoxazole 76.^77^ (Scheme 24).

**Scheme 25:**
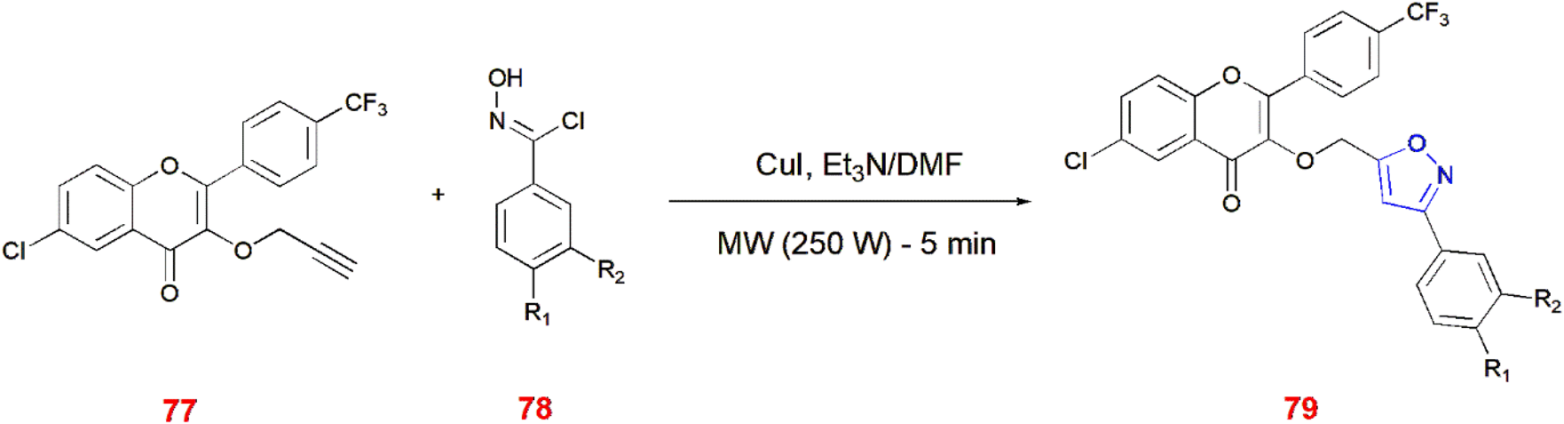
Microwave synthesis of trifluoromethylated flavonoid-based isoxazoles 79.

Trifluoromethylated flavonol 77 on treatment with aromatic oxime 78 in the presence of triethylamine and CuI in DMF generated flavonoids with isoxazole ring 79. These reactions were carried out *via* microwave irradiation (250 W) for 5 min.^78^ (Scheme 25).

### 2.4 Cycloisomerization

**Scheme 26:**
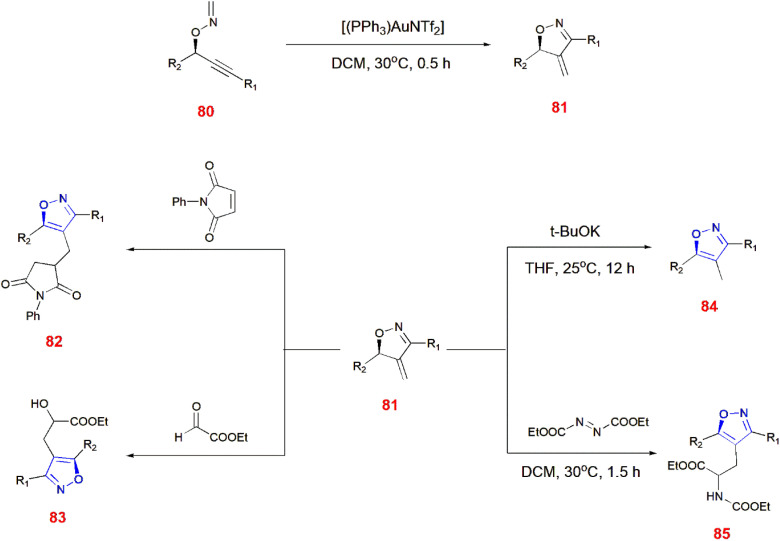
Synthesis of isoxazole derivatives 82–85.

**Scheme 27:**
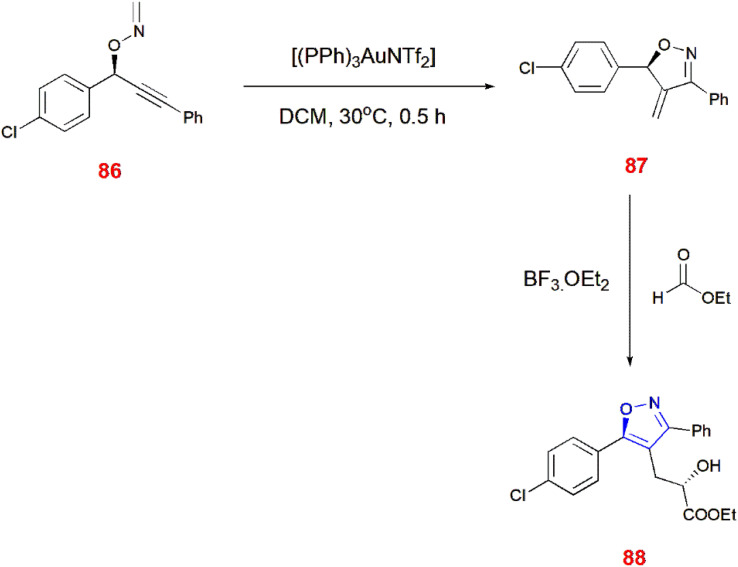
Synthesis of isoxazole analogue 88.

Nakamura and co-workers illustrated the preparation of 4-methylated isoxazoline analogues 81 by the rearrangement of *O*-propargylic formaldoxime 80*via* intermolecular methylene group transfer reaction using gold catalyst. Later, ene or isomerization reaction of isoxazolines 81 takes place with maleimide, glyoxalate, potassium *tert*-butoxide and azodicarboxylate to yield corresponding isoxazole derivatives 82–85.^79^ (Scheme 26) Similarly, chiral isoxazoles 88 can be made from *O*-propargylic oxime 86 facilitated through chirality transfer. Furthermore, the chirality of *O*-propargylic oxime was retained without any changes leading to the formation of isooxazoline derivatives 87 which undergoes treatment with glyoxalate in the presence of boron trifluoride etherate to give expected chiral isoxazole 88.^80^ (Scheme 27).

**Scheme 28:**
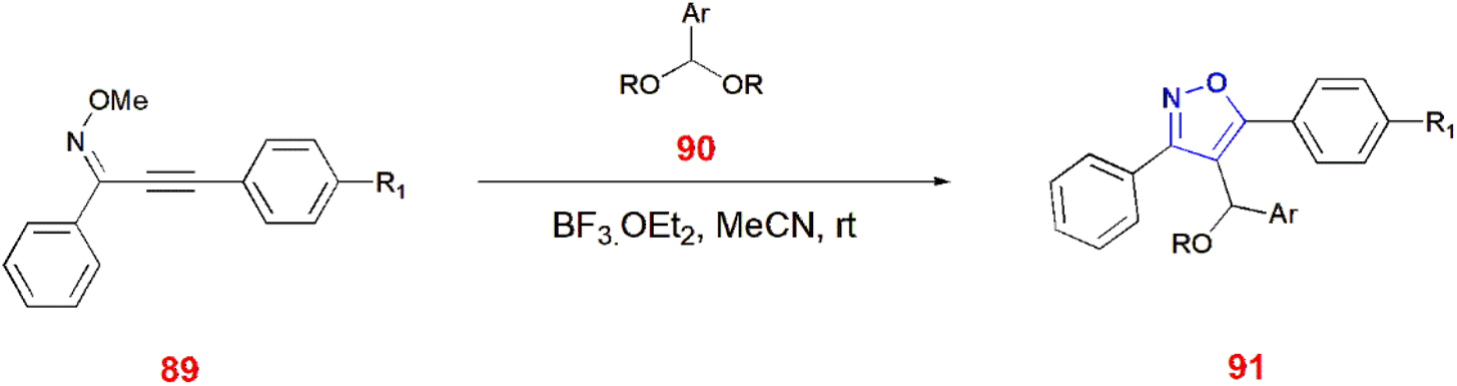
Synthesis of 4-[alkoxy(aryl)methyl]-substituted isoxazoles 91.

4-[Alkoxy(aryl)methyl]-substituted isoxazoles 91 were synthesized from aryl acetals 90 and alkynyl-*O*-methyl oximes 89. This is promoted by the oxocarbenium cations, which cyclize alkynyl-*O*-methyl oximes 89 intramolecularly. For this occurrence, triple bond of alkynyl-*O*-methyl oxime 89 must be activated by oxocarbenium cations generated from aryl acetals 90 in the presence of boron trifluoride etherate, acetonitrile at room temperature for 10–15 min which led to the formation of isoxazoles 91*via* intramolecular 5-endo cyclization.^81^ (Scheme 28).

**Scheme 29:**
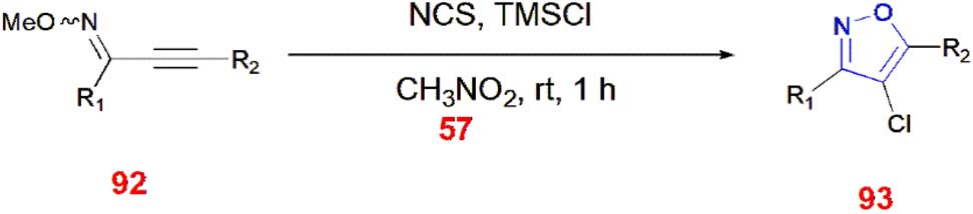
Synthesis of 4-chloroisoxazoles 93.

Chlorinative cyclization of NCS and trimethylsilyl chloride led to the formation of 4-chloroisoxazoles 93. (*E*/*Z*)-alkynyl-*O*-methyl oximes 92 were isomerized to its *Z* form to cyclize isoxazole 93 in the presence of nitromethane 57 at room temperature for 1 h.^82^ (Scheme 29).

### 2.5 Direct functionalization

**Scheme 30:**
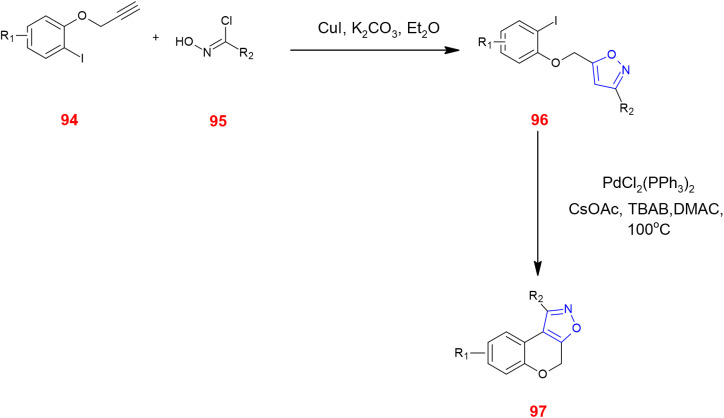
Synthesis of tricyclic fused isoxazoles 97*via* direct functionalization.

The direct functionalization of the isoxazole ring at C-3, C-4 and C-5 is important because it is labile under basic conditions. This is accomplished either by C–H activation or transition metal cross coupling reactions. Propargyl phenyl ethers 94 were made to react with hydroxymoyl chloride 95 in the presence of CuI and potassium carbonate in diethyl ether to give isoxazole 96. This intermediate was then cyclized using Pd(ii) complex generating tricyclic fused isoxazoles 97.^83^ (Scheme 30).

**Scheme 31:**
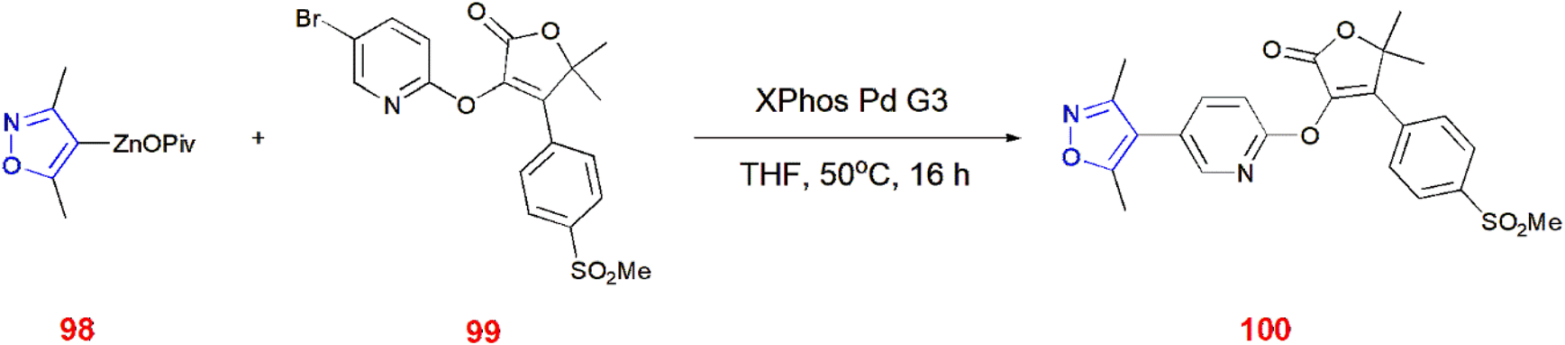
Negishi cross coupling of isoxazole zinc pivalates 98.

Negishi coupling of isoxazole zinc pivalates 98 with bromopyridine derivative 99 which is highly functionalized in the presence of the XPhos Pd G3 catalyst, produced the drug-like scaffold 100. The reactivities of organozinc pivalates can be determined from several experimental methods.^84^ (Scheme 31).

**Scheme 32:**
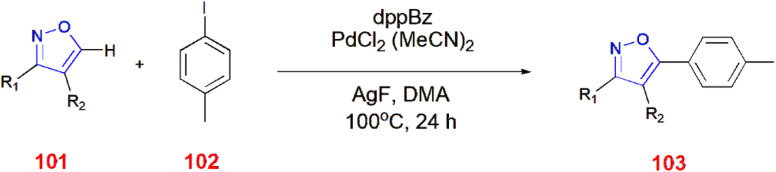
C–H arylation of 3,4-disubstituted isoxazoles 101.

3,4-Disubstituted isoxazoles 101 underwent C–H arylation *via* 1,2-bis(diphenylphosphino)benzene (dppBz), which facilitated the coupling at C-5 of the isoxazole ring with aryl iodides 102 to yield coupled product 103.^85^ (Scheme 32).

**Scheme 33:**
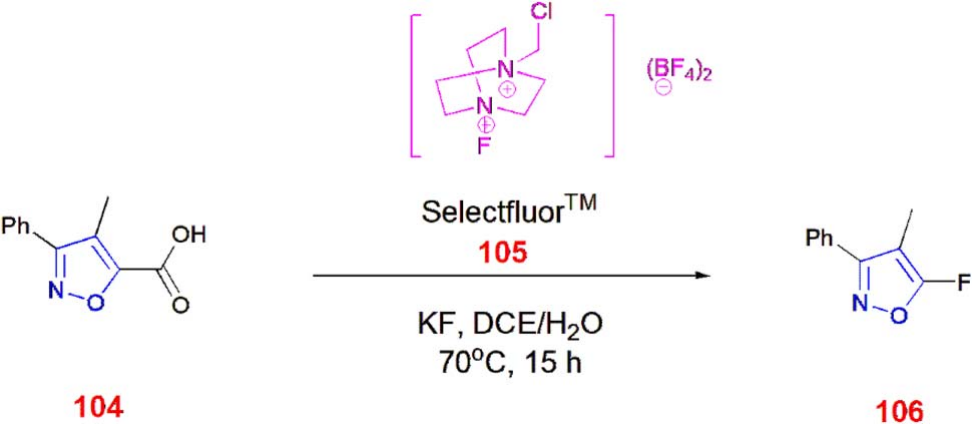
Fluorination of isoxazole acids 104*via* decarboxylation.

**Scheme 34:**
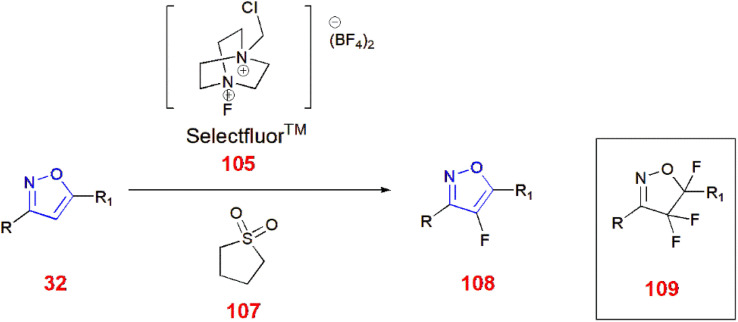
Fluorination of 3,5-disubstituted isoxazoles 32.

Tang *et al.*, demonstrated various methods of fluorinating isoxazole acids 104 directly by decarboxylation in the presence of KF in 1,2-dichloroethane/water at a ratio of 2 : 1 at 70 °C for 15 h using Selectfluor™ 105 to yield fluorinated isoxazole 106 ^86^ (Scheme 33). Moreover, Selectfluor™ 105 was also used to fluorinate 3,5-disubstituted isoxazoles 32 at the C-4 position to give 108 in modest yields. Excess use of Selectfluor™ gave isoxazolines 109 with difluoro and monofluoro substituents at C-4 and C-5, respectively.^87^ (Scheme 34).

**Scheme 35:**
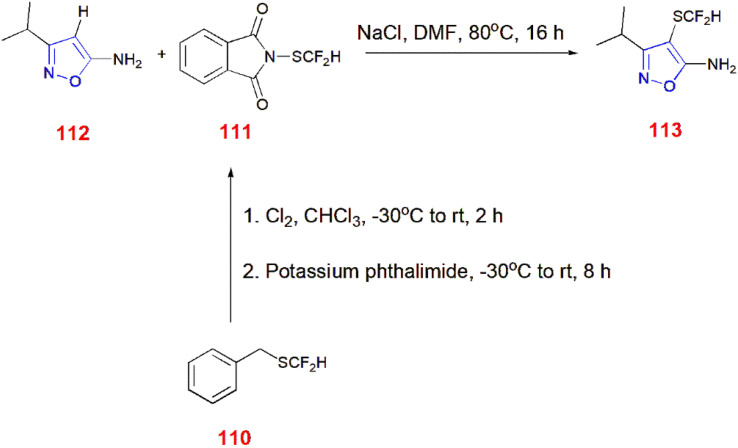
Difluoromethylthiolation of 5-aminoisoxazole 112.

5-Aminoisoxazole 112 underwent difluoromethylthiolation using *N*-difluoromethylthiophthalimide 111 to give 113. *N*-difluoromethylthiophthalimide 111 was prepared from reacting benzyl difluoromethylthioether 110 with chlorine in chloroform to generate dichlorocarbene at −30 °C to room temperature for 2 h. Furthermore, potassium phthalimide was added to give the desired reagent 111.^88,89^ (Scheme 35).

## 3 Biology of isoxazoles

### 3.1 Antibacterial and antifungal activity

Antibacterial agents fall under the class of antimicrobials that can be bacteriostatic^90^ or bactericidal^91^ in nature. Thus, several commercially available drug molecules containing isoxazoles have been approved for the treatment of acute bronchitis^92^ and infections in the urinary tract.^93^ However, isoxazole-containing drugs such as oxacillin,^94^ flucloxacillin,^95^ dicloxacillin,^96^ cloxacillin,^97^ sulfamethoxazole^45^ and sulfisoxazole,^98,99^ which are commercially available, are listed in Table 2.

**Table 2:** Commercially available isoxazole-containing antibacterials

Name	Structure	Action
Oxacillin	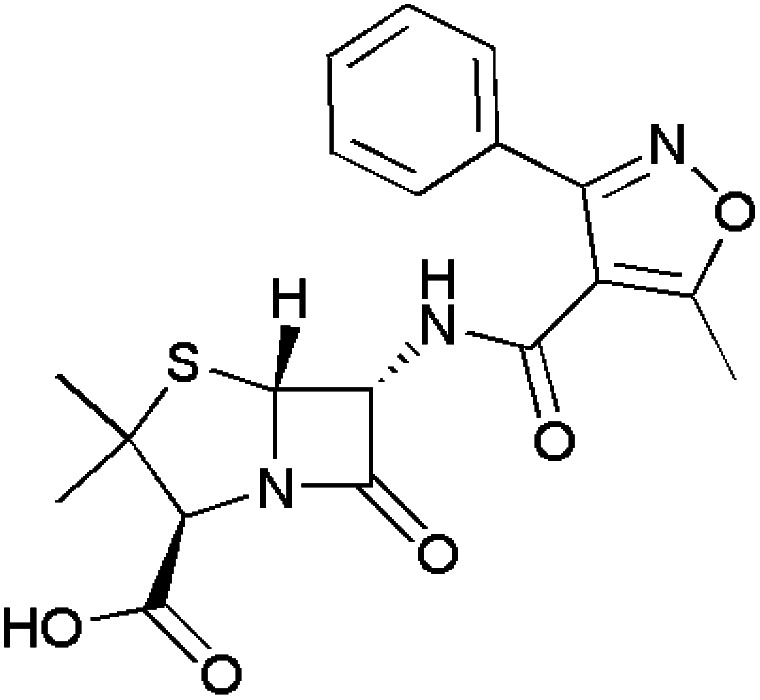	Resistant to staphylococci infections
Flucloxacillin	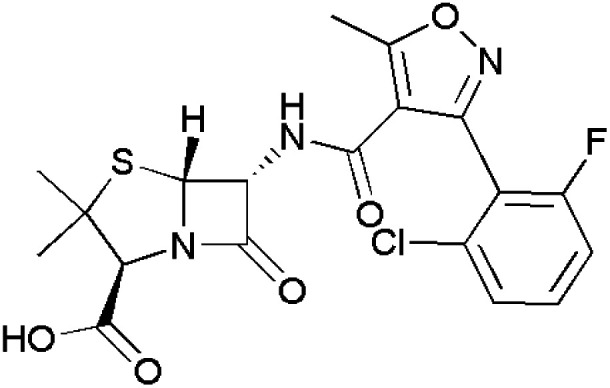	Used in the treatment of pneumonia and endocarditis
Dicloxacillin	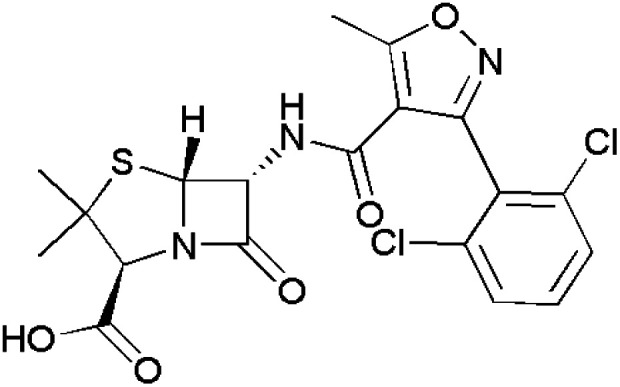	Acts upon penicillin resistant-staphylococci infections
Cloxacillin	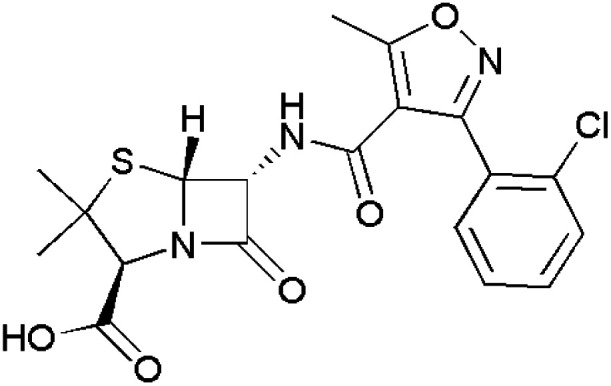	Resistant to streptococcal, pneumococcal and staphylococcal infections
Sulfamethoxazole	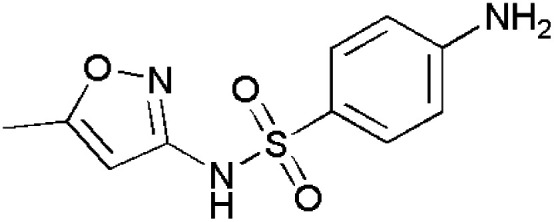	Used to treat urinary tract and gastrointestinal tract infections
Sulfisoxazole	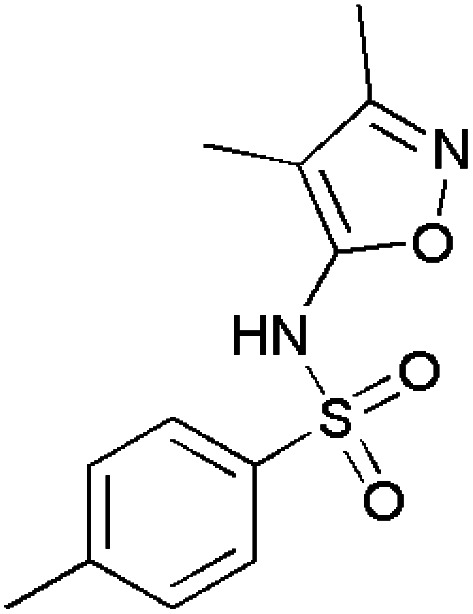	Used to treat meningitis, inclusion conjunctivitis

Raju *et al.*, synthesized novel urea and thiourea derivatives of 6-fluoro-3-(piperidin-4-yl)benzo[*d*] isoxazole and evaluated their antimicrobial activity keeping tetracycline as the standard reference drug. Among the six derivatives, compounds 111–113 showed their inhibition against *Bacillus subtilis* and *Staphylococcus aureus.* Furthermore, 111 and 113 showed additional inhibition against *Escherichia coli* and *Pseudomonas aeruginosa*. Structure–activity relationship (SAR) studies revealed that the presence of electron withdrawing groups like trifluoro and chloro increased the activity to a greater extent.^100^

Khazi *et al.*, synthesized new sulfones and sulfides of methylene-bridged benzisoxazolylimidazo[2,1-*b*][1,3,4]thiadiazoles, tested them against *E. coli* ATCC 35218 and *B. subtilis* ATCC 6633 and concluded that most had significant to modest activity when compared with the standard drug ampicillin. Compounds 114–117 showed good activity against these strains. Additionally, compounds 115–117 showed activity against fungal species such as *Candida albicans* and the activity of 115 was almost equivalent to the standard drug Clotrimazole. Furthermore, compounds 114 and 118 showed good inhibitory effect against *Aspergillus fumigatus*. Coumarin and methoxy substituents made these compounds more active against fungal and bacterial strains. Especially, the delocalization of π-electrons in 114 and 115 resulted in penetration into lipid membranes as they are lipophilic in nature.^101^

**Fig. 2:**
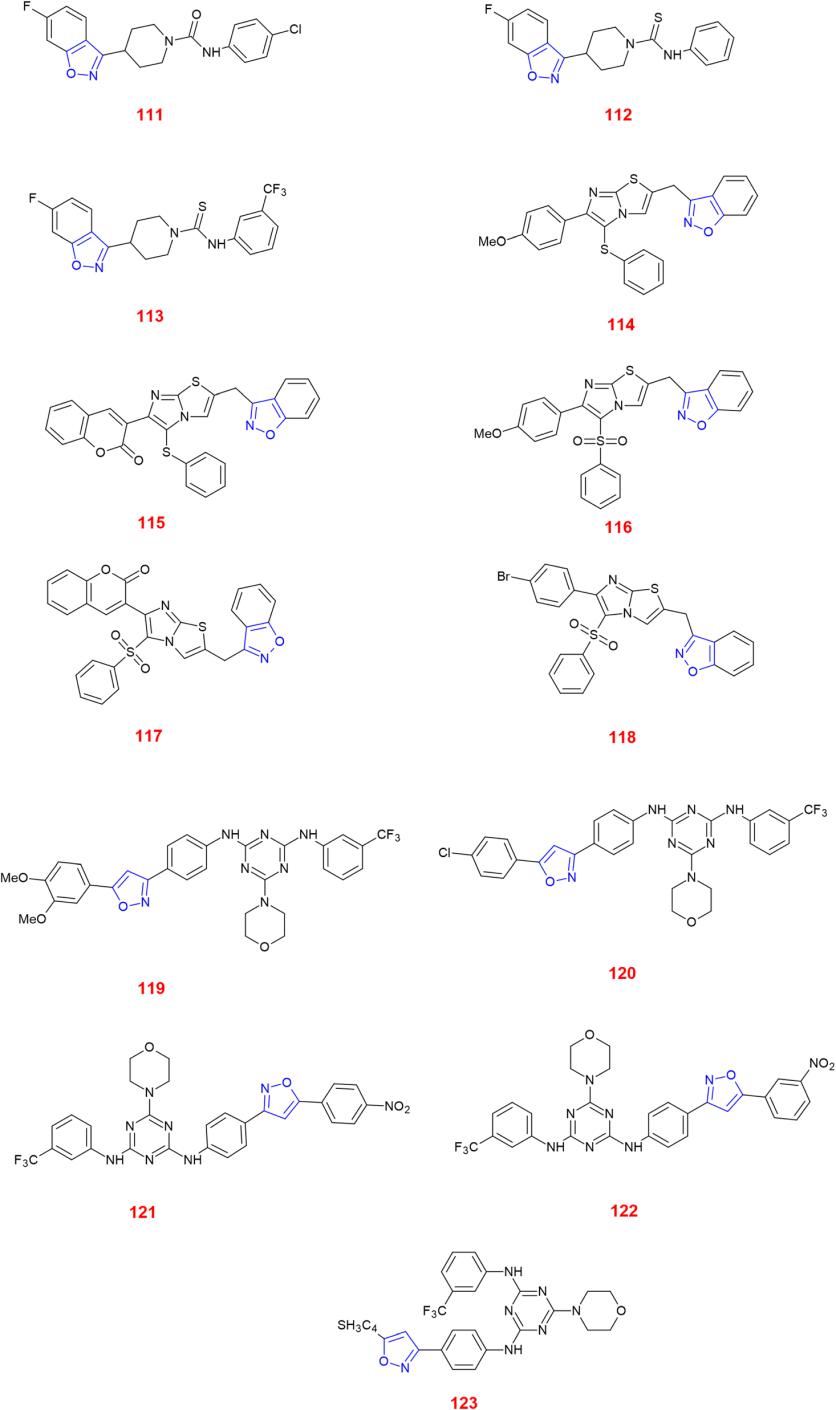
Isoxazole-containing antibacterial and antifungal compounds.

Anjani *et al.*, prepared novel derivatives of 1,3,5-triazines containing aminopyridines, isoxazoles and acetyl pyrazoline moieties, checked for their antimicrobial proficiency and determined their minimum inhibitory concentration (MIC). Compound 119 was effective against *S. aureus* bearing MIC value of 100 μg ml^−1^, quite good when compared with ampicillin with 250 μg ml^−1^ but not better than chloramphenicol and ciprofloxacin, both with MIC of 50 μg ml^−1^. Compounds 121 and 123 both showed MIC values of 100 and 125 μg ml^−1^ against *Streptococcus pyogenes*, which is equivalent to ampicillin with MIC 100 μg ml^−1^ but moderate towards chloramphenicol and ciprofloxacin with 50 μg ml^−1^. When Gram-negative bacteria such as *E. coli* is taken, compound 122 showed MIC value of 100 μg ml^−1^ which was again equivalent to ampicillin but was quite higher than chloramphenicol and ciprofloxacin having MIC of 50 and 25 μg ml^−1^, respectively. Compounds 121 and 122 showed MIC values of 200 μg ml^−1^ same as that of ampicillin against *S. aureus*. Notably, compound 122 showed great antifungal activity against *C. albicans* with MIC value of 250 μg ml^−1^. Compounds 119–121 showed same activity as that of standard drug griseofulvin showing MIC value of 500 μg ml^−1^. However, the SAR studies revealed that the presence of CF_3_ in the compounds 119–123 made them active as antimicrobial scaffolds. Among them, 119 and 123 showed highest antimicrobial activity. Such electronic changes or modifications enable molecules to increase the binding energy with the microbial target site, thereby enhancing potency.^102^ (Fig. 2).

### 3.2 Anticancer activity

Isoxazoles have good anticancer activities and can be used to treat various carcinomic conditions. Several commercially available isoxazole-containing drug molecules such as acivicin,^103^ XN05,^104,105^ PNZ5,^106^ NVP-AUY922 ^107–109^ shown in Table 3.

**Table 3:** Commercially available isoxazole-containing anticancer drugs with their biological action

Name	Structure	Action
Acivicin	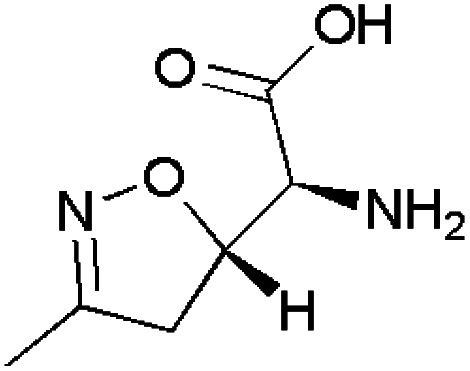	Cancer biomarker
XN05	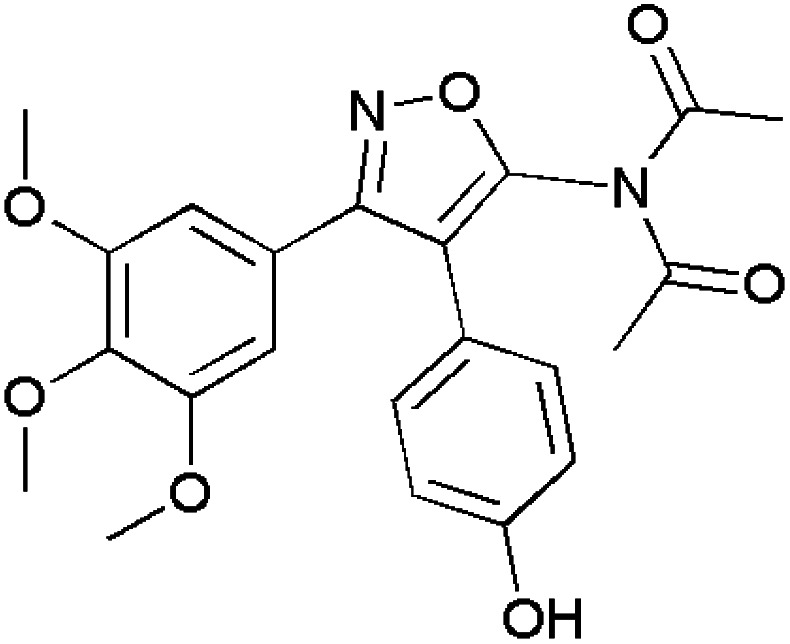	Acts against hepatocellular cancer
PNZ5	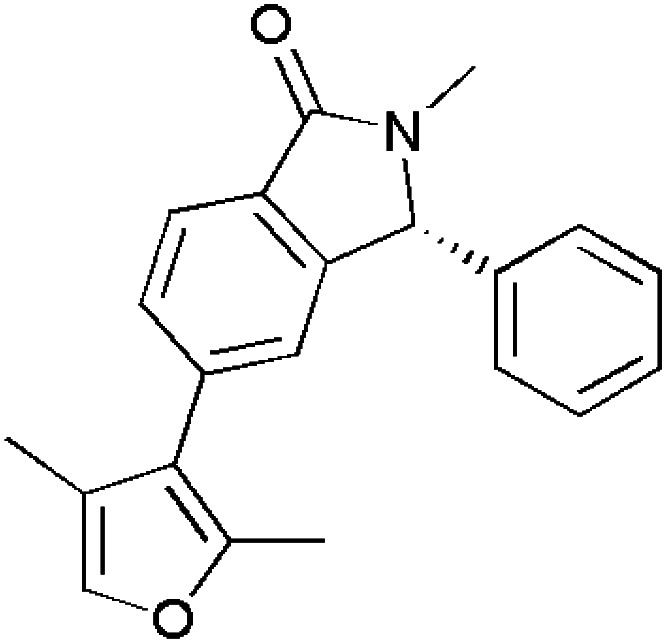	Inhibits gastric cancer cell growth
NVP-AUY922	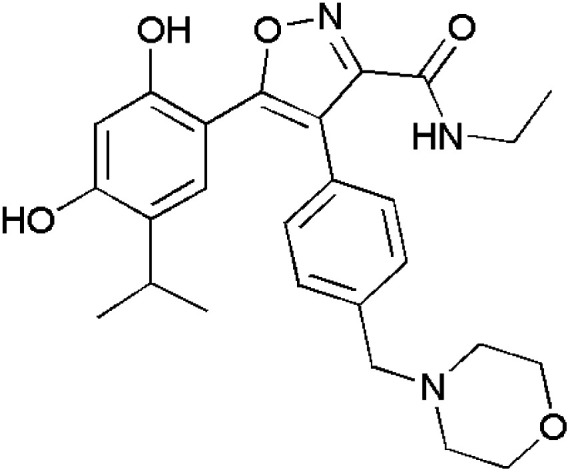	Acts against breast cancer

Eid *et al.*, synthesized and evaluated the biological performance of new isoxazole–amide analogues. As a result of the anticancer evaluation, these derivatives were tested against HeLa, Hep3B, and MCF-7 cell lines, their IC_50_ (half-maximal inhibitory concentration) values were compared with that of doxorubicin. It was found that, compound 124 was most active against HeLa cell line with IC_50_ value of 15 : 48 ± 0 : 89 μg ml^−1^. However, compound 125 was considerably active against HeLa showing IC_50_ value of 18 : 62 ± 0 : 79 μg ml^−1^. Compounds 124 and 126 showed anticancer activity against Hep3B cell line with IC_50_ 23 : 98 ± 1 : 83 μg ml^−1^ and 23 : 44 ± 1 : 99 μg ml^−1^, respectively.^110^

Hawash *et al.*, synthesized and studied the biological activity of phenyl-isoxazole–carboxamide analogues. These derivatives were tested for their anticancer profiles against HeLa, MCF-7, Hep3B, HepG2, and Hek293T cell lines comparing with those of doxorubicin. Compounds 127–129 showed quite good anticancer activity against Hep3B cell lines showing the IC_50_ values (μM) of 5.96 ± 0.87, 6.93 ± 1.88 and 8.02 ± 1.33, respectively. Compound 130 was active against the MCF-7 cell line with IC_50_ value (μM) of 4.56 ± 2.32. Compound 129 showed excellent anticancer activity against HeLa cell line with IC_50_ value (μM) of 0.91 ± 1.03.^111^

Ketan and co-workers produced some isoxazole derivatives with phenyl rings linked with a diazo group and evaluated their anticancer activity against PC3 and HEK normal cell lines. Compounds 131–134 showed anticancer activity against PC3 among which 134 was highly potent. However, these compounds showed anticancer activity only with high doses (640 μM) in normal cell lines. The SAR studies revealed the significance of the electron-withdrawing groups such as –F, –Cl, and –Br. The *ortho*-substituted bromo compound 134 demonstrated valuable cytotoxic effects than the rest of the halogen substituted analogues.^112^

Jarina *et al.*, generated novel isoxazole derivatives, tested their anticancer activity against MCF-7 cell lines and reported their IC_50_ values (μg ml^−1^). Among the six derivatives, compounds 135 and 136 showed good anticancer activity reflecting IC_50_ values (μg ml^−1^) of −26.32 and −29.57 comparing with that of standard drug Adriamycin. Moreover, molecular docking studies demonstrated the binding affinity of −9, favouring hydrophobic binding interactions with topoisomerase II.^113^

**Fig. 3:**
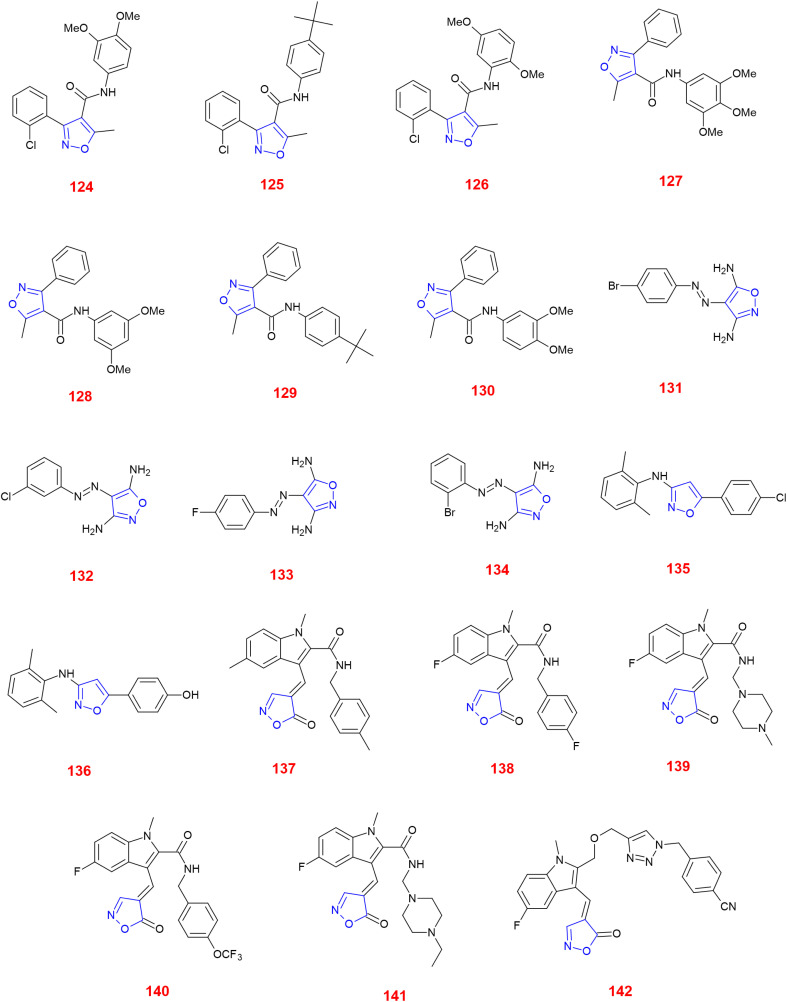
Isoxazole analogues showing anticancer activity.

Panathur *et al.*, produced isoxazoles linked with indole and studied their antiproliferation activity against MCF-7 and HT-29 cell lines comparing with Gemcitabine. Compounds 137 and 138 were effective against MCF-7 cell lines showing IC_50_ values (μM) 7.72 and 5.51. Compounds 139–142 were tested against HT-29 cell lines and showed interesting IC_50_ values (μM) −4.82, −2.59, −4.80 and −4.83, respectively. The docking analysis proved that compound 140 containing a trifluoromethyl benzyl ether linkage was responsible for the increased cytotoxicity and is one of the lead compounds that could furnish significant anti-proliferative activity along with SIRT1 enzyme.^114^ (Fig. 3).

### 3.3 Anti-inflammatory activity

Isoxazoles are reported to have good anti-inflammatory activities and they control inflammation as there is a great need to reduce or treat edema. Normally, they follow two pathways namely, the cyclooxygenase (COX) and lipoxygenase (LOX) pathways.^115–117^ Some of the isoxazole-containing drugs such as Parecoxib,^118^ Valdecoxib,^119,120^ Mofezolac,^121,122^ Leflunomide^123^ are shown in Table 4.

**Table 4:** Commercially available isoxazole containing anti-inflammatory drugs

Name	Structure	Action
Parecoxib	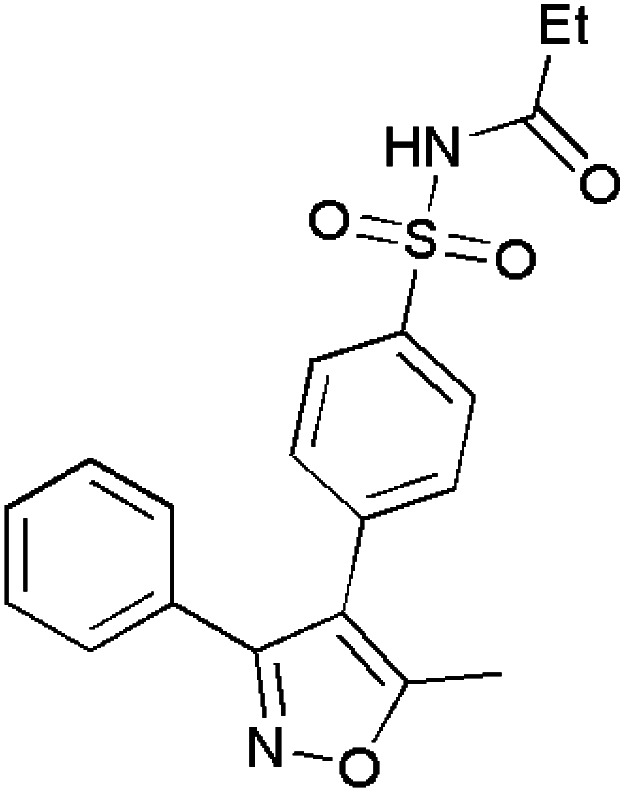	COX-2 inhibitor
Valdecoxib	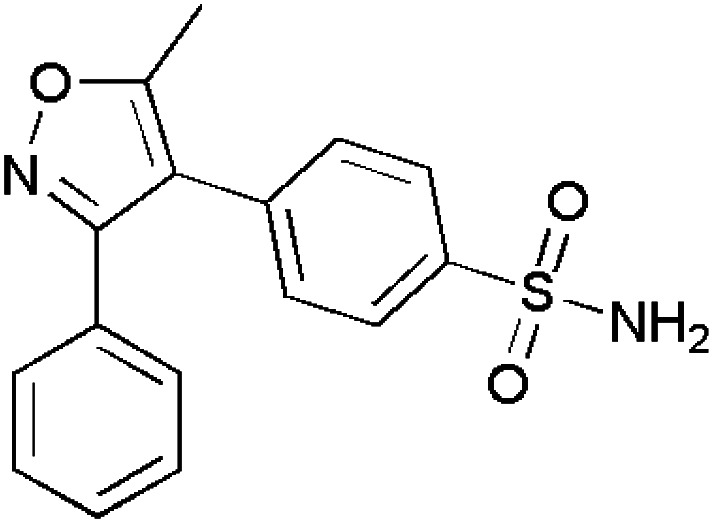	Relieves arthritic inflammation
Mofezolac	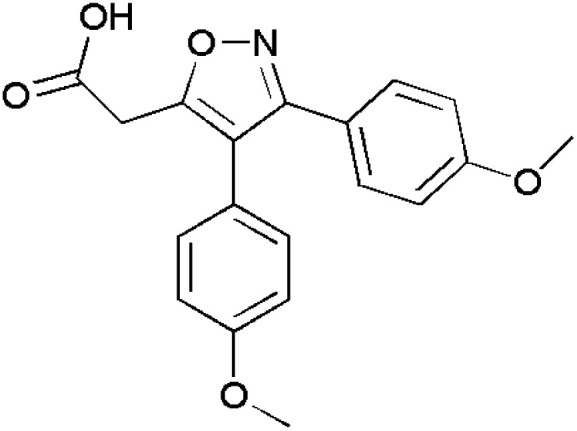	Controls inflammation in rheumatoid arthritis
Leflunomide	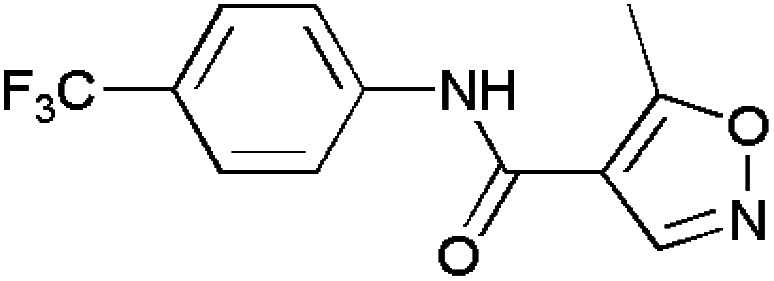	Blocks formation of DNA thereby reducing inflammation in arthritic patients

Abdellal worked on synthesizing novel isoxazoles derivatives and checked for their anti-inflammatory activity using rat paw edema model induced by carrageenan^124^ as a suitable domain to perform experiments. Among various isoxazoles, compound 143 showed the ED_50_ (mg kg^−1^) value 45 when compared to standard drug bearing 40. While, other compounds 144 and 145 showed ED_50_ (mg kg^−1^) value in the range of 48–50. Thus, compound 143 was closer and more effective in reducing inflammation in rat paws. The molecular docking scores with the range −14.77 to −15.63 kcal mol^−1^ demonstrated the high selectivity of compounds 143–145 towards COX-2 receptor site and revealed well-established bonding interaction with active pharmacophores such as :O: of C=O and :N of isoxazole ring.^125^

Bhupinder Kumar *et al.*, synthesized indole-linked isoxazoles and tested their anti-inflammatory activity using the most accepted paw edema model. After injecting the rats with carrageenan to develop edema, these compounds were tested and the changes in the volume of paw edema at 1 h, 2 h and 4 h intervals, respectively. After thorough examination, compound 146 showed highest anti-inflammatory activity with 77.42% reduction after 4 h. Compounds 147 and 148 also showed good anti-inflammatory by reducing paw edema by 67.74 and 61.29%. Apart from these three compounds, there was an interesting result with respect to compound 149 which showed anti-inflammatory activity at 2 h interval and reduced in later hours which the biologists suggested the methoxy group at meta position had affected for such kind of deviation by inactivating the compound. However, the strong evidence was laid from the SAR studies which demonstrated the presence of 4-methoxy and 3-methoxy in compounds 147 and 148 favoured COX-2 inhibitory, anti-inflammatory and analgesic action. The selectivity index for COX-2 was highest with methoxy group at 4th position and resulted in slight decrease with the shift to 3rd position.^126^

Khanum *et al.*, produced few novel 3-phenyl-5-furan isoxazole derivatives and evaluated their anti-inflammatory activity. Among the synthesized derivatives, compound 150 exhibited potency in inhibiting COX-2 with IC_50_ value (μM) 9.16 ± 0.38 in relation with the reference drugs indomethacin and diclofenac sodium. Through molecular docking studies, compounds 151–154 showed their affinity towards COX-1 rather than COX-2. For exhibiting COX-2 affinity, it is very essential for the isoxazole substrate to be hydrophobic and compound 150 had two chloro groups at *ortho* and *para* positions which makes them to interact with the active site. Apart from COX-2, it has also shown good 15-LOX inhibition with IC_50_ value (μM) 8.15 ± 0.16. These inhibitions are the great evidence for them to act as anti-inflammatory agents and lead molecules for the development of newer drug molecules.^127^

**Fig. 4:**
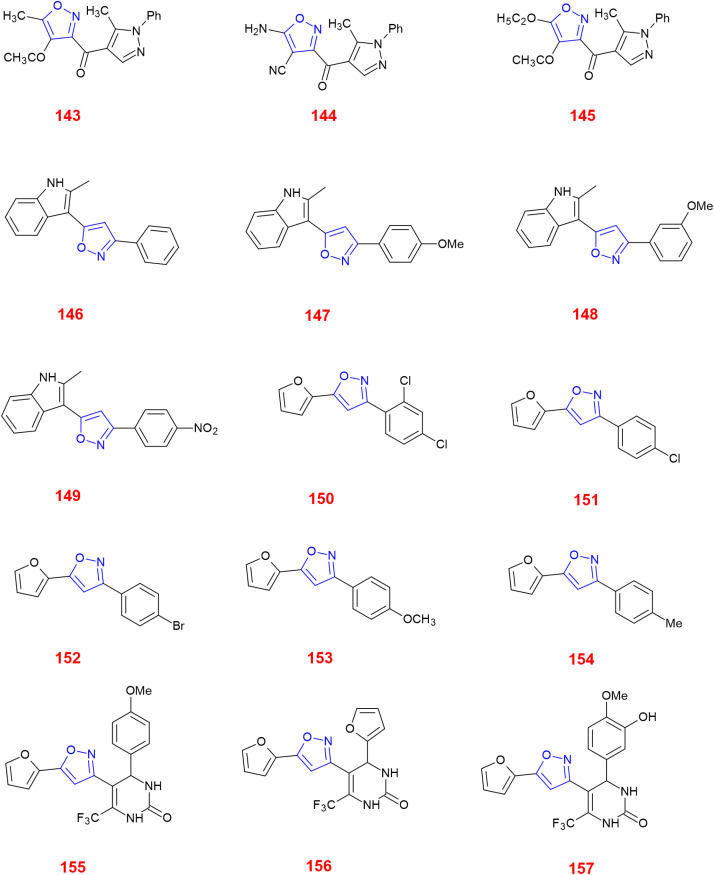
Isoxazole derivatives showing anti-inflammatory activity.

Khan *et al.*, gave some isoxazole derivatives and carried out 5-LOX inhibitory assay for the designed compounds. Compound 155 showed promising 5-LOX inhibition with IC_50_ value 3.67 μM. Additionally, compounds 156 showed good 5-LOX inhibition next to compound 155. Compound 156 and 157 showed promising results in COX-2 inhibition.^128,129^ (Fig. 4).

### 3.4 Antioxidant activity

Hawash and co-workers determined the potential of novel isoxazole derivatives with respect to their antioxidant capacity. The derivatives of fluoro-phenyl-carboxamide were synthesized previously and then checked for their antioxidant properties using 2,2-diphenyl-1-picrylhydrazyl (DPPH) assay. Trolox was used as the control for the experiments. Its IC_50_ value (μg ml^−1^) was found to be 3.10 ± 0.92. After evaluation, compounds 158 and 159 showed potent antioxidant activity with IC_50_ values (μg ml^−1^) 0.45 ± 0.21 and 0.47 ± 0.33, respectively. Usually, antioxidant compounds exhibit radical scavenging properties wherein the radical will abstract a proton from the antioxidant scaffold. As there are different substituents on the phenyl ring, compound 158 has *tert*-butyl group, whose absence showed very less antioxidant properties. Thus, it was made clear that the presence of electron donating groups were responsible for exhibiting antioxidant properties.^130^

Chalka *et al.*, investigated the antioxidant activity of novel functionalized isoxazoles using DPPH method of screening, taking BHT as the reference standard having IC_50_ value (μg ml^−1^) 28.81 ± 1.84. Among the synthesized isoxazoles, compound 160 showed great antioxidant property with IC_50_ value (μg ml^−1^) of 63.51 ± 1.80. Other than this compound, some other compounds 161–163 exhibited free radical scavenging characteristics having IC_50_ values (μg ml^−1^) of 79.85 ± 1.90, 83.69 ± 1.92 and 87.76 ± 1.94, respectively. The remaining isoxazoles showed moderate DPPH activity. However, the ‘drug-likeness’ evaluation suggested that compounds 161 and 163 were closely adhering to the Lipinski's ‘Rule of Five’ and compound 163 persisted significant oral bioavailability threshold (>55% F). Erstwhile, all compounds 160–164 validated Egan's rule for balanced consideration of lipophilicity and molecular weight.^131^

Nagaraju *et al.*, screened quinazolinone-based isoxazole derivatives for antioxidant activity using DPPH assay. Ascorbic acid was used as the control for the experiments. It was found that, compound 164 exhibited best activity with inhibitory concentrations 1.28 ± 0.33 and 1.39 ± 0.38 μM. However, compound 165 showed significant activity succeeding the prior compound with inhibitory concentrations 2.72 ± 0.34 and 2.78 ± 0.41 μM. Furthermore, these compounds 164 and 165 were evaluated against NADP oxidase as it plays a key role in generating reactive oxygen species. As a result of this, compound 164 best fitted into the groove of NADP oxidase with quite acceptable ADME properties without violating Lipinski's rule, exploring the potential in developing into therapeutic agent.^132^

**Fig. 5:**
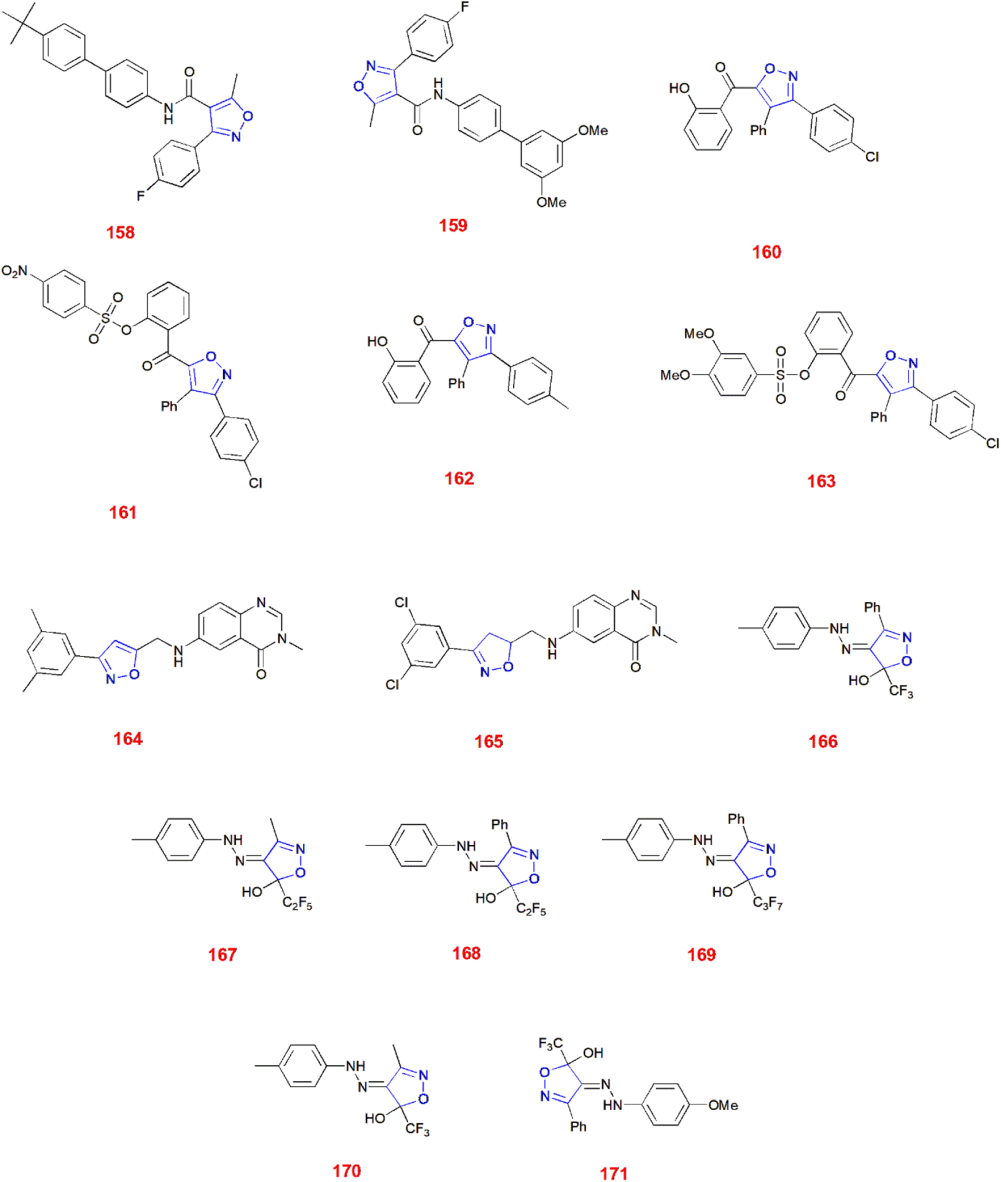
Isoxazole derivatives showing antioxidant activity.

Victor and co-workers synthesized and studied the antioxidant properties of 4-arylhydrazinylidene-isoxazoles containing polyfluoroalkyl groups. 2,2′-Azino-bis(3-ethylbenzothiazoline-6-sulfonic acid) (ABTS)^133,134^ and ferric reducing antioxidant power (FRAP)^135,136^ free radical scavenging assays were carried out for evaluating antioxidant properties taking Trolox as the reference standard with Trolox equivalent antioxidant capacity (TEAC) 1. Compounds 166–169 were found to be lead compounds in ABTS assay having TEAC 1.50 ± 0.07, 1.80 ± 0.06, 2.00 ± 0.10 and 1.90 ± 0.09, respectively. Compounds 170 and 171 were found to be leads for FRAP assay as they effectively reduced the Fe^3+^ complex.^137^ (Fig. 5).

### 3.5 Antitubercular activity

*Mycobacterium tuberculosis* causes Tuberculosis, an air-borne lung infection *i.e.*, contagious in nature.^138^*Mycobacterium tuberculosis* falls under the three major classes of the genus *Mycobacterium* that cause tuberculosis, leprosy and other non-tuberculous mycobacteria.^139–141^ Thus, many compounds were developed to treat tuberculosis among which isoxazole containing molecules have gained great attention and importance. Quinoline–isoxazole containing compounds have been widely studied and some of these include compounds 172–176.^142–146^

Sharabu and Pappula synthesized and studied the antitubercular activity of isoxazole clubbed pyrimidine derivatives. *Mycobacterium tuberculosis* H37Rv was used for this study and Pyrazinamide was used as the reference antitubercular drug and was carried out using dilution method at various concentrations ranging from 0.78 to 100 μg ml^−1^. Compound 177 and 178 showed excellent activity at MIC 0.78 μg ml^−1^. Compounds 179–183 showed better activity with MIC 1.56 μg ml^−1^ than the reference drug (MIC 3.125 μg ml^−1^).^147^

Marco Pieroni and others evaluated the antitubercular activity of 5-(2-aminothiazol-4-yl)isoxazole-3-carboxamides taking Streptomycin and Isoniazid as reference standards and REMA method for determining MIC toward the tubercular strain. *M. tuberculosis* H37Rv strain was used for this study and it revealed that compounds 184 and 185 showed maximum antitubercular activity with MIC (μg ml^−1^) 1.0 and 0.5, respectively.^148^

**Fig. 6:**
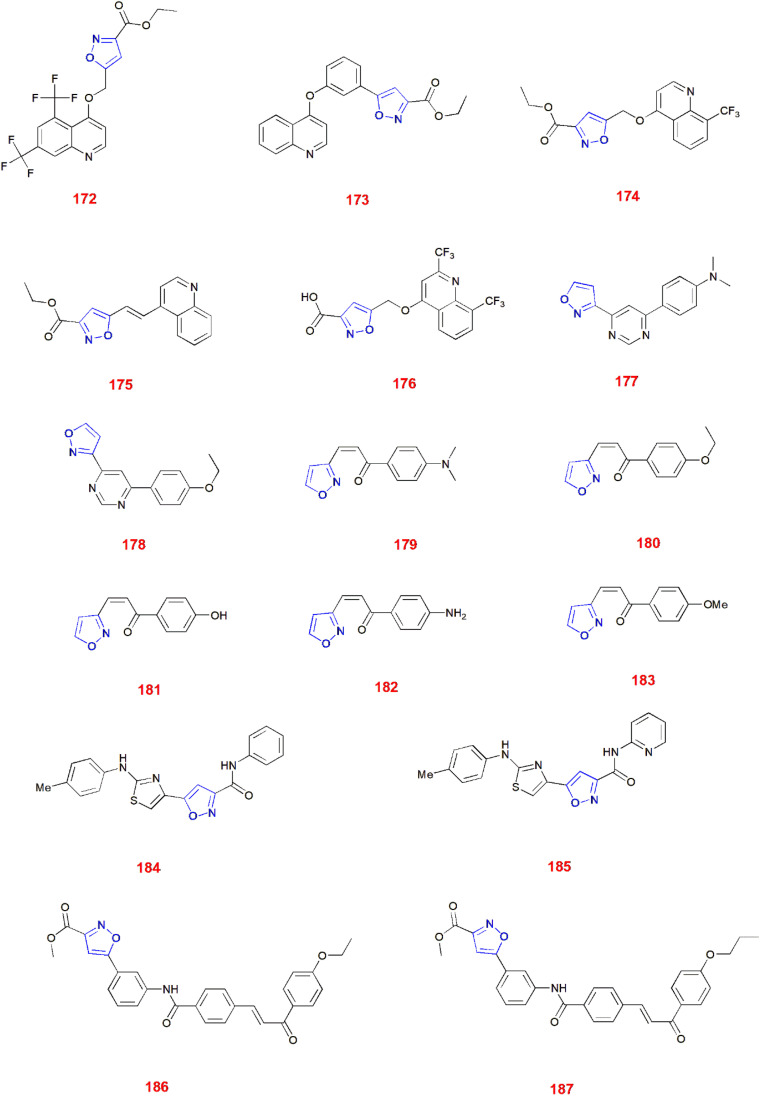
Isoxazoles analogues showing antitubercular activity.

Sahoo *et al.*, synthesized several novel chalcone linked 5-phenyl-3-isoxazolecarboxylic acid methyl esters and tested for their antitubercular activity against *M. tuberculosis* H37Rv strain taking isoniazid, streptomycin, rifampicin and ethambutol as reference drugs. After careful examination, they concluded that compound 186 and 187 were very potent antitubercular agents with MIC (μg ml^−1^) 0.25 and 0.12, respectively.^149^ (Fig. 6).

## 4 Conclusion

Isoxazoles are synthesized *via* unique reactions such as cycloadditions and the inclusion of click reactions makes them more attractive. The green synthesis of isoxazoles has also attained increased attention, as the environment is a major concern for chemists working on molecules worldwide. The direct functionalization of isoxazoles illustrates the basics of organic reactions and how unique they are upon arylation. In addition to synthetic methods, isoxazoles are also important medicinally. The biological implications of isoxazoles have made them more attributable to clinical dimensions. Most of the isoxazole analogues discussed in this review were successful in inhibiting various bacterial and fungal strains that cause various infections and other health-related diseases. However, such derivatives were also effective with anti-proliferative action when tested against various cancer cell lines such as HeLa, MCF-7, Hep3B, PC3, HEK, HT-29 *etc.* Such findings accomplish the potential of the isoxazole cores as anticancer agents with the existing standard anticancer drugs. The ability of isoxazoles to inhibit cyclooxygenase (COX) and lipoxygenase (5-LOX) pathways led for the strong anti-inflammatory action. The scope of isoxazoles is further broadened by their remarkable antioxidant and antitubercular actions supported by the ‘drug-likeness’ evaluation. Some isoxazole-containing compounds have shown even better results than the standard reference drugs which are commercially available. Thus, isoxazoles can be fascinating molecules in the world of organic chemistry for chemists to work in academic research and pharmaceutical research and development.

## Abbreviations

NCS: *N*-Chlorosuccinimide
NIS: *N*-Iodosuccinimide
DCM: Dichloromethane
DCE: Dichloroethane
THF: Tetrahydrofuran
DME: Dimethyl ether
ACN: Acetonitrile
DMF: Dimethylformamide
TBAB: Tetrabutylammonium bromide
DMAC: Dimethylacetamide
K-PHI: Potassium poly(heptazine imide)
DABCO: 1,4-Diazabicyclo[2.2.2]octane
TMSN_3_: Trimethylsilyl azide
TMSCl: Trimethylsilyl chloride
DBU: 1,8-Diazabicyclo[5.4.0]undec-7-ene
MW: Microwave
*m*-CPBA: *meta*-Chloroperbenzoic acid
TFA: Trifluoroacetic acid
SAR: Structure–activity relationship
ATCC: American type culture collection
MIC: Minimum inhibitory concentration
HeLa: Henrietta lacks
MCF-7: Michigan cancer foundation-7
PC3: Human prostate cancer cell line
IC_50_: Half-maximal inhibitory concentration
ED_50_: Median effective dose 50
HEK: Human embryonic kidney cell line
SIRT1: Silent information regulator sirutin1
COX: Cyclooxygenase
LOX: Lipooxygenase
DNA: Deoxyribonucleic acid
DPPH: 2,2-Diphenyl-1-picrylhydrazyl
NADP: Nicotinamide adenine dinucleotide phosphate
ADME: Absorption, distribution, metabolism, excretion
ABTS: 2,2′-Azino-bis(3-ethylbenzothiazoline-6-sulfonic acid)
FRAP: Ferric reducing antioxidant power
TEAC: Trolox equivalent antioxidant capacity
REMA: Resazurin microtiter assay plate

## Ethical statement

This study doesn't involve the use of any humans or animals.

## Data availability

All data has been obtained from peer-reviewed articles cited in the reference list, with no additional datasets utilized.

## Author contributions

Glanish Jude Martis: writing – original draft, software. Santosh L. Gaonkar: writing – review & editing, supervision.

## Conflicts of interest

On behalf of the authors, the corresponding author declare no interests.
